# Acute sensitization of the P3 event-related potential response to beverage images and the risk for alcohol use disorder

**DOI:** 10.1016/j.addicn.2022.100041

**Published:** 2022-10-28

**Authors:** Roberto U. Cofresí, Thomas M. Piasecki, Bruce D. Bartholow

**Affiliations:** a Department of Psychological Sciences, University of Missouri, Columbia, MO 65211, United States; b Center for Tobacco Research and Intervention and Department of Medicine, University of Wisconsin, Madison, United States

**Keywords:** Alcohol, P3, LPP, Incentive salience, Cue-reactivity, Subjective response

## Abstract

Previous research suggests the amplitude of the P3 event-related potential (ERP) response reflects the incentive value of the eliciting stimulus, and that individuals with trait-like lower sensitivity (LS) to the acute effects of alcohol, a potent risk factor for alcohol use disorder (AUD), tend to show exaggerated P3 ERP responses to alcohol beverage cues (compared to their peers with higher sensitivity; HS). No prior research has examined trajectories of the cue-elicited P3 response across repeated trials of nonreinforced cue presentations. Characterizing these trajectories can be informative as to potential mechanisms linking LS with increased AUD risk. Here, we tested whether individual differences in alcohol sensitivity are associated with different trial-by-trial trajectories of the P3 elicited by alcohol and nonalcohol reward cues (infrequent oddball/target stimuli) using a large sample of emerging adults (*M*_age_ = 19.53; *N* = 287; 55% female; 86% White; 90% right-handed) stratified for alcohol sensitivity. Multilevel models adjusted for age, sex, handedness, and alcohol use indicated that: (i) the P3 response to alcohol and nonalcohol reward cues alike sensitized (i.e., increased) across trials; (ii) across the task, the P3 response to alcohol cues was larger for the LS than the HS phenotype; and (iii) the P3 difference score (alcohol - nonalcohol) was larger for the LS than HS phenotype only across the first half of task. Findings suggest that whereas incentive value attribution may be a mechanism for alcohol cue-triggered attentional biases for both LS and HS individuals, LS individuals more consistently over-attribute incentive value to alcohol cues.

## 1. Introduction

### 1.1. P3/LPP response to alcohol/drug cues as an indicator of alcohol/drug incentive value

A growing body of work suggests that individual differences in neurocognitive responses elicited by alcohol/drug-related cues, including selective attention [19, 46, 66, 79] and motivational significance [18, 21, 25, 31, 58], may index susceptibility to alcohol/drug incentive sensitization [50], one of the neuropathophysiological processes theorized to drive disordered alcohol and drug use behavior. The incentive sensitization theory of addiction (ISTA; [8,72]) posits a vicious cycle in which repeated alcohol/drug use induces neuroadaptations that progressively render certain vulnerable individuals hyper-reactive (“sensitized”) to the incentive-motivational value of alcohol/drug-predictive cues, such that the latter more powerfully capture attention and impel approach, thereby increasing the frequency and/or quantity of alcohol/drug use.

Among the best characterized neurophysiological indicators of incentive-motivational value attribution to an eliciting stimulus is the amplitude of the mid-to-late latency positive components of the event-related potential (ERP) such as the P3 and late positive potential (LPP). Indeed, both the P3 and LPP have been shown to index the extrinsic (top-down) and/or intrinsic (bottom-up) incentive-motivational significance of eliciting stimuli (e.g., [6,27,77]). Although the P3 and LPP were discovered and described in different domains of cognitive neuroscience research (e.g., the P3 in canonical cognitive tasks employing simple stimuli with short durations [≤ 1 s], the LPP in affective picture viewing tasks employing complex stimuli with longer durations [e.g., 2–4 s]), they appear to index a largely overlapping set of higher-order neurocognitive operations or processes related to stimulus significance (for review, see: [37]), with the P3 corresponding to the early window of the LPP [26, 28, 89]. Importantly, enhanced amplitude P3/LPP response to alcohol/drug cues relative to control cues has been associated with heavier alcohol/drug use cross-sectionally [39, 46, 66] and prospectively [3, 4]. The amplitude of the P3/LPP response to alcohol/drug cues may even be able to differentiate individuals with alcohol/drug use disorders from those without [58,60,71]. Hence, there is growing interest in its clinical utility [11, 41].

Many prior studies have reported an enhanced amplitude P3/LPP response to alcohol cues among individuals with heavier or more hazardous alcohol use (e.g., [39, 46, 55, 66]), but others have not (e.g., [43,88]). Studies from our laboratory suggest a more nuanced story. Specifically, we have found that enhanced amplitude P3 response to alcohol cues is not related to heavier alcohol use *per se* but rather to trait-like low sensitivity (LS) to the acute effects of alcohol [5, 10, 25, 54], a proposed endophenotype that confers risk for alcohol use disorder (AUD) [45, 62, 69, 75].

Much remains unknown, however, concerning the *nature* of differences in P3/LPP responses to alcohol/drug-related cues within and between individuals. Traditional P3/LPP component scoring involves averaging the P3/LPP response elicited by each individual stimulus presentation in a task. While useful for increasing the signal-to-noise ratio in the ERP (see [52]), this signal averaging approach rests on two related assumptions that might not hold in all situations (see [86]): (1) that the ERP signal of interest is constant across trials, and, therefore, (2) that any trial-by-trial variation in the ERP signal merely reflects noise contributed by momentary lapses of attention, stimulus misperception, and/or non-learning processes (e.g., sensory adaptation, response fatigue). Moment-to-moment changes in affective/motivational states as well as learning processes that accrue across trials (e.g., habituation, sensitization) can result in meaningful variation in ERP signals, thereby undermining the validity of signal averaging in some situations. Indeed, prior research has shown that, with appropriate modeling, meaningful trial-by-trial variability in ERP responses can be derived (see [74, 85]).

There is good reason to believe that the P3/LPP response elicited by visual alcohol-related cues in typical laboratory paradigms might not be uniform across trials. Typical human laboratory cue reactivity paradigms are *de facto* Pavlovian cue extinction (viz., non-reinforcement) procedures, in which a previously conditioned cue is repeatedly presented in the absence of the cue-predicted outcome (i.e., reward; see [20]). Repeated presentation of any stimulus generally results in habituation of stimulus-elicited autonomic, attentional, and behavioral responses (see [20]). Thus, to the extent that the P3/LPP response to alcohol/drug-related images—which ostensibly are naturally conditioned visual cues signaling availability of the depicted reward—reflects an attentional orienting response [50], its magnitude should diminish (habituate) across non-reinforced cue presentations within a given measurement occasion. In keeping with this possibility, within-session habituation of the P3 response has been demonstrated in traditional target-detection oddball paradigms [34, 68] as well as for the LPP response to affective pictures [10, 13, 22]. Whether higher-risk and lower-risk drinkers experience differential habituation of P3/LPP responses to alcohol-related cues during laboratory cue-reactivity paradigms has not been examined.

Several possible patterns or trajectories of alcohol cue-elicited P3/LPP responses that might differentiate higher- and lower-risk drinkers should be considered. First, the enhanced mean P3/LPP response among higher-risk drinkers could reflect a consistently elevated level of response across repeated trials (i.e., a difference in intercept that is maintained across cue presentations). A second possibility—not necessarily independent of the first—is that the enhanced P3/LPP response among high-risk drinkers reflects resistance to habituation or extinction of the response across trials. This could occur if higher- and lower-risk drinkers experience different reinforcement schedules with respect to alcohol cue conditioning. For example, if higher-risk drinkers are more likely to select environments where visual alcohol-related stimuli are more frequently encountered (e.g., bars/pubs, parties, peer groups) but do not always seek or consume alcoholic beverages in response to cues in those environments, then cue-alcohol associations are on a partial reinforcement schedule for them. Preclinical studies have shown that animals on partial reinforcement schedules are more resistant to extinction of conditioned cue responses compared to animals on continuous reinforcement schedules (e.g., [87]). Furthermore, preclinical studies have shown that animals that “sign-track” (i.e., attribute incentive salience to reward-predictive cues) are more resistant to extinction of conditioned cue responses than are animals that “goal-track” (i.e., learn the reward-predictive value of cues but do not attribute incentive salience to those cues) [1, 23]. To the extent that LS drinkers’ cue-reactivity shares features with sign-tracking, as we have proposed (see [14,25]), their P3/LPP responses to alcohol-related cues also might resist habituation.

A third possibility is that the enhancement of the alcohol cue-elicited P3/LPP response observed in at-risk drinkers reflects an exaggerated *initial* response to cue exposure yet masks an accelerated habituation of responses to subsequent cue exposure. This could occur if higher- and lower-risk drinkers differ in the extent of their natural histories of cue-alcohol conditioning. For example, assuming that they are similarly exposed to visual alcohol-related cues in everyday life (e.g., ads online, commercials on TV or streaming platforms, signage and stock at grocery and convenience stores), higher-risk drinkers may be more likely to “act on” their conditioned cue responses than lower-risk drinkers. That is, they may be more likely to seek and purchase or consume alcohol beverages in response to those cues. If so, then they are undergoing more extensive cue-alcohol conditioning than lower-risk drinkers. Consistent with this possibility, preclinical studies have shown that animals that have undergone more extensive appetitive conditioning are *more* sensitive to the extinction of conditioned cue responses than animals that have undergone less conditioning (e.g., [80, 81]), presumably due to a more intense cued expectancy violation effect (viz., greater prediction error) among individuals for whom the cue has almost always accurately predicted reward receipt.

A fourth possibility is that the enhancement of the alcohol cue-elicited P3/LPP response observed in at-risk drinkers reflects sensitization of the cue-elicited response across trials. This seems unlikely, given that human cue-reactivity tasks typically do not involve delivery of reinforcing stimuli following cue presentation, and therefore growth in the P3/LPP amplitude across trials cannot be due to within-task reinforcement learning *per se*. However, it remains possible that growth in the P3/LPP response could occur due to acute sensitization of arousal state systems^1^ driven by two design features in alcohol/drug cue-reactivity paradigms: (i) the more arousing nature (i.e., higher affective intensity) of alcohol/drug-depicting images relative to control images depicting affectively neutral objects or scenes (see [70]); and (ii) the use of a low presentation frequency for alcohol/drug images relative to control images. This combination—high stimulus intensity and low frequency of presentation—is one of few known to result in sensitization of stimulus-elicited responses (see [36]). Nonetheless, response sensitization across trials within a single measurement occasion is rarely observed outside of specific laboratory paradigms (e.g., fear-potentiated startle; [35, 49, 56, 82]).

### 1.2. The current study

The current study examines whether individual differences in alcohol sensitivity determine the trial-by-trial trajectory of the P3 response to visual cues for alcohol reward in a large, nonclinical sample of alcohol-using emerging adults. We advanced the following hypotheses.

Hypothesis 1: The P3 response to alcohol cues, non-drug ingested reward cues, and control non-reward cues alike will diminish (habituate/extinguish) across repeated non-reinforced cue presentations.
Hypothesis 2: The P3 response to alcohol cues will be larger among individuals reporting lower compared to higher sensitivity to the acute effects of alcohol.
Hypothesis 3: The P3 response to alcohol cues *relative to* nondrug ingested reward cues or control (non-ingested, nondrug) cues will be larger among individuals reporting lower compared to higher sensitivity to the acute effects of alcohol.
Hypothesis 4: The P3 response to alcohol cues will diminish (habituate/extinguish) across repeated non-reinforced cue presentations less rapidly among individuals reporting lower compared to higher sensitivity to the acute effects of alcohol.

## 2. Method

### 2.1. Participants

Data in this report are from a large, multi-method study examining the link between individual differences in alcohol sensitivity, alcohol use and alcohol cue reactivity across late adolescence to early emerging adulthood. Underage drinkers from the community completed an online eligibility screening survey and were invited to the laboratory if they were age 18–20 years, reported at least monthly alcohol use in the past year and one binge-drinking episode (4+/5+ drinks in 2 h for females/males, respectively) in the past 6 months, and reported no history of neurological disease, head injury, or unsuccessful attempts to reduce alcohol use. See Supplemental Information for recruitment strategies and detailed inclusion-exclusion criteria. Eligible individuals were invited to enroll strategically to stratify the sample for biological sex, alcohol sensitivity, and alcohol use. The current analyses draw on data from the first of three laboratory sessions (*N* = 318).^2^ Data from four participants were excluded because their EEG could not be segmented (event markers were not recorded) and from 27 participants whose EEG data contained fewer than 20 artifact-free segments per condition.^3^ Table 1 presents characteristics for the final analytic sample (*N* = 287).

### 2.2. Materials

#### 2.2.1. Self-report measures

##### Alcohol sensitivity.

Participants completed the 15-item Alcohol Sensitivity Questionnaire (ASQ) [24, 63], which queries the number of drinks a respondent must consume to experience various subjective effects from drinking alcohol. More positive ASQ scores indicate *lower* alcohol sensitivity and predict *higher* subjective stimulation, *lower* subjective sedation, and *lower* subjective intoxication during laboratory alcohol challenge [24]. ASQ scores were standardized to reduce bias [48] and stratified by sex to avoid confounding with sex differences in alcohol pharmacokinetics [29]. Full details are given in Supplemental Information. ASQ scores exhibited excellent internal consistency (*α* = .95). Descriptive statistics are presented in Table 1; associations with alcohol use are given in Table S1.

##### Alcohol use.

Participants completed questionnaire measures of past-year typical frequency (drinking days per week), typical quantity (drinks per drinking day), maximum quantity of alcohol consumed within 24 hr, and of binge-drinking episodes per week in the past 6 months [61] (see Table 1). Participants also indicated age at first intoxication and age at onset of regular drinking. AUD symptoms also were assessed using the Mini International Neuropsychiatric Interview (MINI) AUD module [78]. Full details and scaling are given in Supplemental Information. Individual differences in typical alcohol use pattern across the past year were indexed by computing alcohol quantity-frequency (AlcQF) scores, the product of past-year typical use frequency and quantity. Descriptive statistics for all alcohol use measures are presented in Table 1; inter-relationships are given in Table S1.

#### 2.2.2. Oddball picture viewing task

Participants completed an oddball picture viewing task similar to one used in our previous studies [4, 5, 10, 54]. On each of 400 trials, a color photograph was presented centrally. Non-beverage, low arousal, neutral-valence images (e.g., clothing, tools; “Neutral Cue”) from the Internal Affective Picture System (IAPS) [47] comprised 80% of trials (frequent standard/non-target stimuli). Images of alcohol beverages (e.g., beer can, wine glass; “AlcBev”) and nonalcohol drinks (e.g., soft-drink can, juice bottle; “NADrink Cue”) from the “passive” subset of the Amsterdam Beverage Picture Set (ABPS) [67] each comprised 10% of trials (infrequent oddball/target stimuli). Participants were instructed to press one button as quickly as possible when they saw an alcohol beverage, to press a different button when they saw a nonalcohol drink, and to press neither button when they saw anything else. Other technical details are presented in Supplemental Information.

#### 2.2.3. Psychophysiological measures

##### EEG acquisition and processing.

EEG was recorded at 512 Hz from 32 Ag/AgCl electrodes (mastoid reference) arranged in the expanded 10–20 system [2]). Impedance was kept below 10 kΩ. Offline, the EEG was re-referenced to the average of the two mastoids, resampled at 256 Hz, and bandpass filtered (2^nd^ order Butterworth with half-amplitude cut-offs: 0.1–30 Hz) using eeglab [17] and ERPlab [51]. Independent components analysis (ICA) was conducted, and an EEGlab routine was used to identify and remove components corresponding to blinks as well as eye movements and other artifacts [59]. The EEG was then segmented into stimulus-locked epochs and epochs with absent or erroneous responses were discarded. Epoched data were subject to additional artifact detection and rejection routines.^4^ Additional details are presented in Supplemental Information.

##### P3 scoring.

**Fig. 1.**
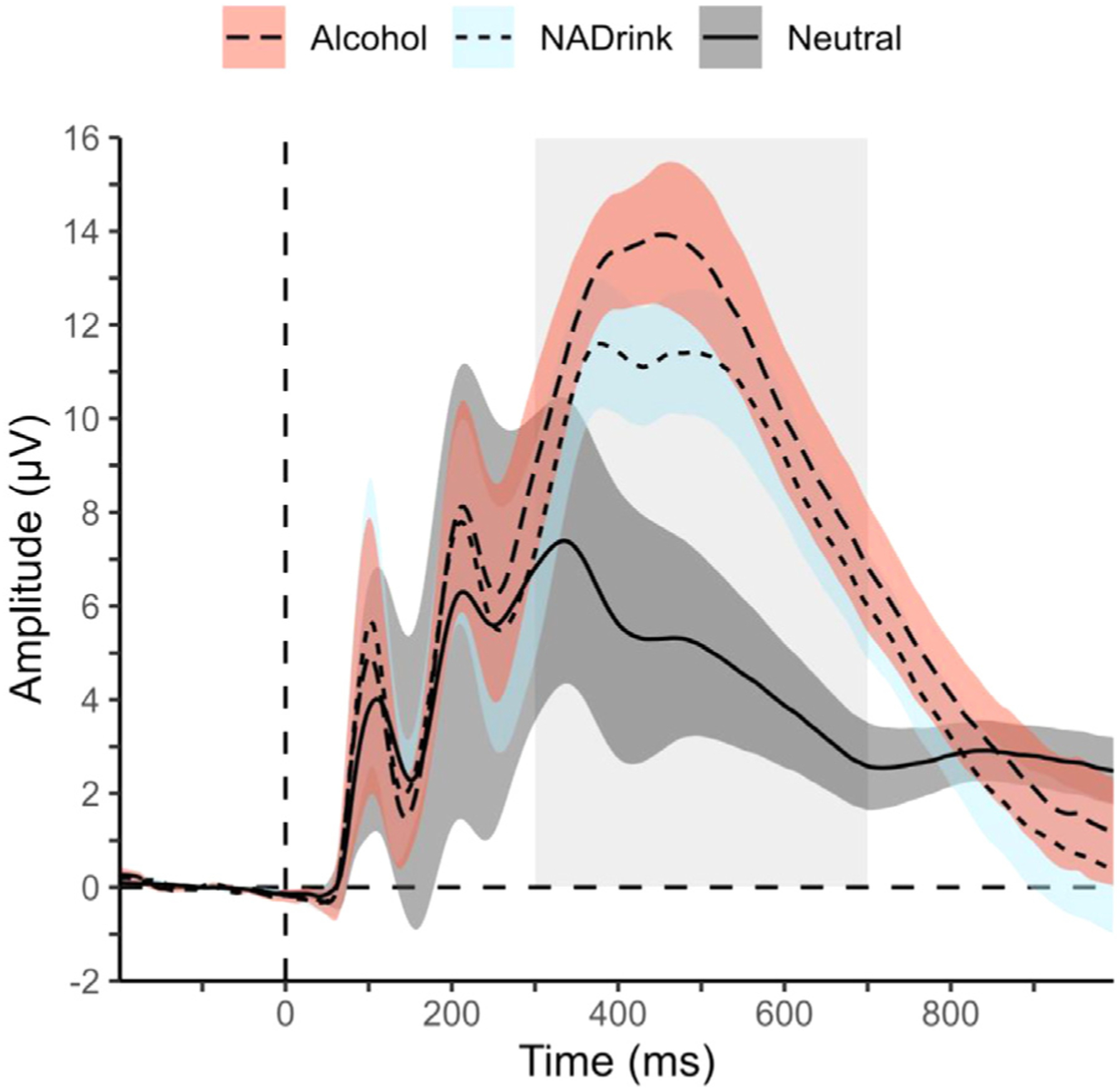
Within-trial timecourse of the event-related potential (ERP) response to alcohol/nonalcohol picture oddball/target stimuli and neutral picture standard/non-target stimuli. Picture onset occurs at 0 ms. Picture offset occurs at 1000 ms. Alcohol = alcohol beverage pictures. NADrink = nonalcohol drink pictures. Neutral = affectively neutral pictures. Thin line at the center of each colored ribbon represents the *M* across participants’ average across 9-electrodes (PZ, P3, P4, P7, P8, PO7, PO8, O1, O2) for the indicated picture type. Ribbon thickness represents ± 1 *SD* across participants. Time-window (300–700 ms) used for P3 mean amplitude measurement is indicated by the lightly shaded rectangular area behind ribbons. Baseline correction was done using the 200 ms before picture onset. Data represents *N* = 287 participants.

P3 mean amplitudes were scored at 9 parietal/occipital electrodes over which P3 amplitude was maximal when image categories were collapsed. Time-windows used for quantification are indicated on the grand average ERP waveforms shown in Fig. 1, and scalp topographies in Fig. S1. P3 scores exhibited excellent internal consistency (*α* = .91–.94), as we and others have shown.

### 2.3. Analytic approach

The overall magnitude and within-task trajectory (linear and quadratic growth) of P3 mean amplitude were analyzed using linear mixed models (LMMs; a.k.a., multi-level models [MLMs]) fit according to best practices [57, 64, 86].^5^ LMMs included both random intercepts for person and electrodes (9 per person) nested within persons as well as random slopes at the person level for all person-centered continuous predictor variables and within-person factors. Image Type was a within-person factor representing the three different trial types (AlcBev, NADrink, Neutral). Linear Time was a person-centered continuous predictor representing trial (i.e., observation) number relative to the total number of experimental trials in the task. Quadratic Time was the square of Linear Time. Since age, sex, and handedness can affect the P3 (e.g., [40]), all were entered as covariates at the person level: age as a sample-centered continuous predictor; sex and handedness as effect-coded categorical predictors. To test hypothesized effects of alcohol sensitivity, ASQ scores were entered as a sample-centered continuous predictor at the person level. To ensure that hypothesized effects of alcohol sensitivity^6^ were not merely a proxy for effects of alcohol use, AlcQF scores also were entered as a sample-centered continuous predictor at the person level.^7^ ANOVA *F*-tests were used to evaluate whether effects significantly contributed to model fit. Regression model summary and ANOVA *F* tables for the alcohol sensitivity hypothesis-testing model, as well as a model ignoring all alcohol-related individual differences, are presented in Supplemental Information. The model was used to estimate covariate-adjusted means across the task while holding ASQ scores at *z* = + 1, which represents extremely low sensitivity (LS) phenotypes, and while holding ASQ scores at *z* = −1, which represents extremely high sensitivity (HS) phenotypes. Pairwise comparisons were conducted on the covariate-adjusted model-estimated means corresponding to the start, middle, and end of the task for these two phenotypes using two-sided asymptotic *z*-tests. The Benjamini-Hochberg false discovery rate adjustment procedure was used to maintain 5% Type 1 error rate across multiple comparisons [7]. The threshold for significance was *p* < .05.

### 2.4. Procedure

Upon arrival, participants provided informed consent and sobriety was verified (breath alcohol concentration = .000 g%). Participants were prepared for EEG recording and then completed the picture viewing task. See Supplemental Information for additional laboratory procedure details.

## 3. Results

**Fig. 2.**
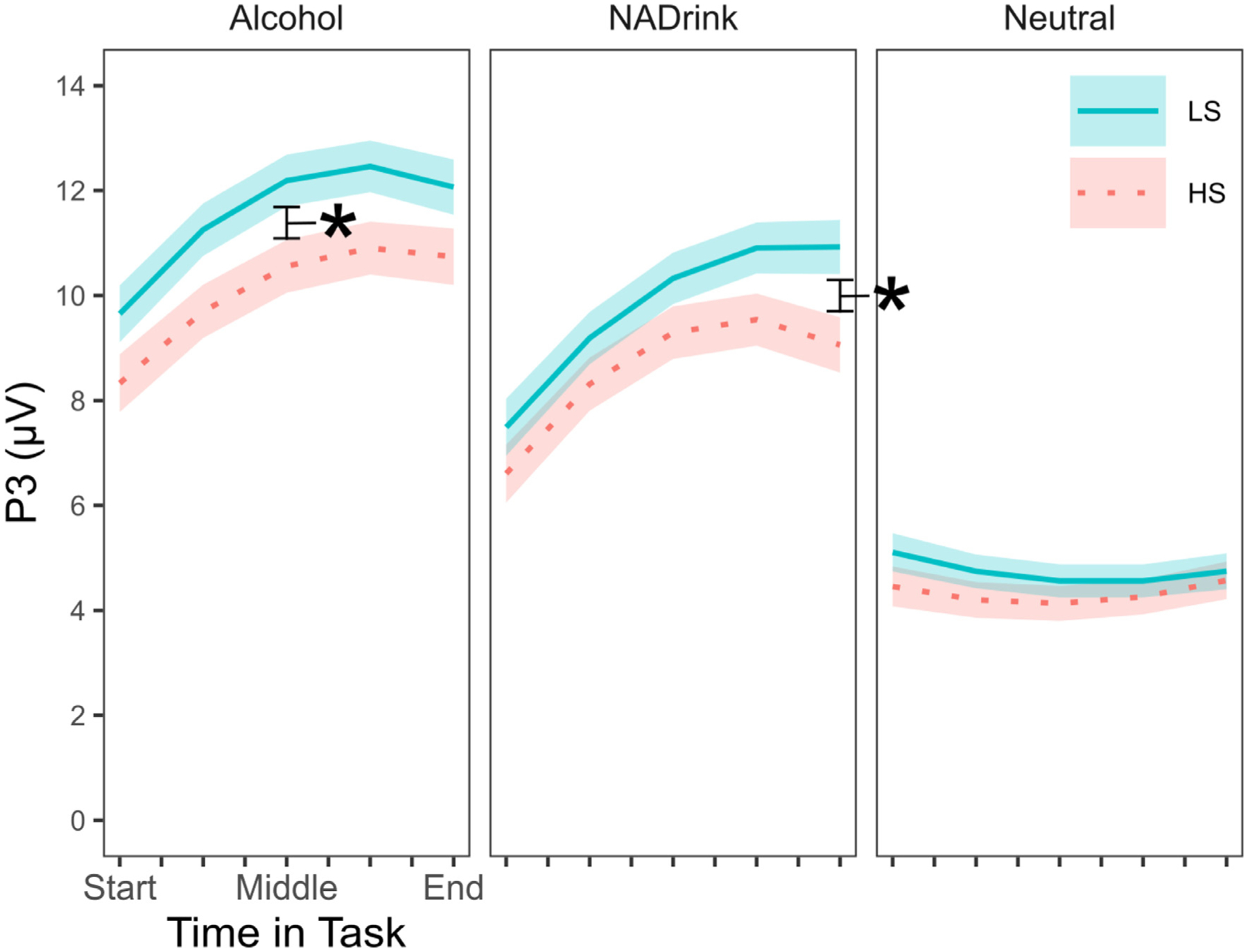
Within-session trajectory of P3 mean amplitude (μV) for alcohol/nonalcohol picture oddball stimuli and neutral picture standard stimuli as a function of alcohol sensitivity. Alcohol = alcohol beverage pictures. NADrink = nonalcohol drink pictures. Neutral = affectively neutral pictures. Start = first artifact-free trial for each person. Middle = artifact-free trial that bisects each person’s set of artifact-free trials. End = final artifact-free trial for each person. Thin line at the center of each colored ribbon represents the covariate-adjusted LMM-estimated *M* P3 score at different relative times in the picture viewing task and the thickness of each colored ribbon represents the covariate-adjusted LMM-estimated ± 1 *SE*. LMM-estimated *M* ± *SE* were derived twice: once holding zASQ at *z* = −1 SD, which corresponds to high sensitivity (HS) phenotypes, and once holding zASQ at *z* = + 1 SD, which corresponds to low sensitivity (LS) phenotypes.* = *p* < .05 for alcohol sensitivity phenotype comparison.

**Fig. 3.**
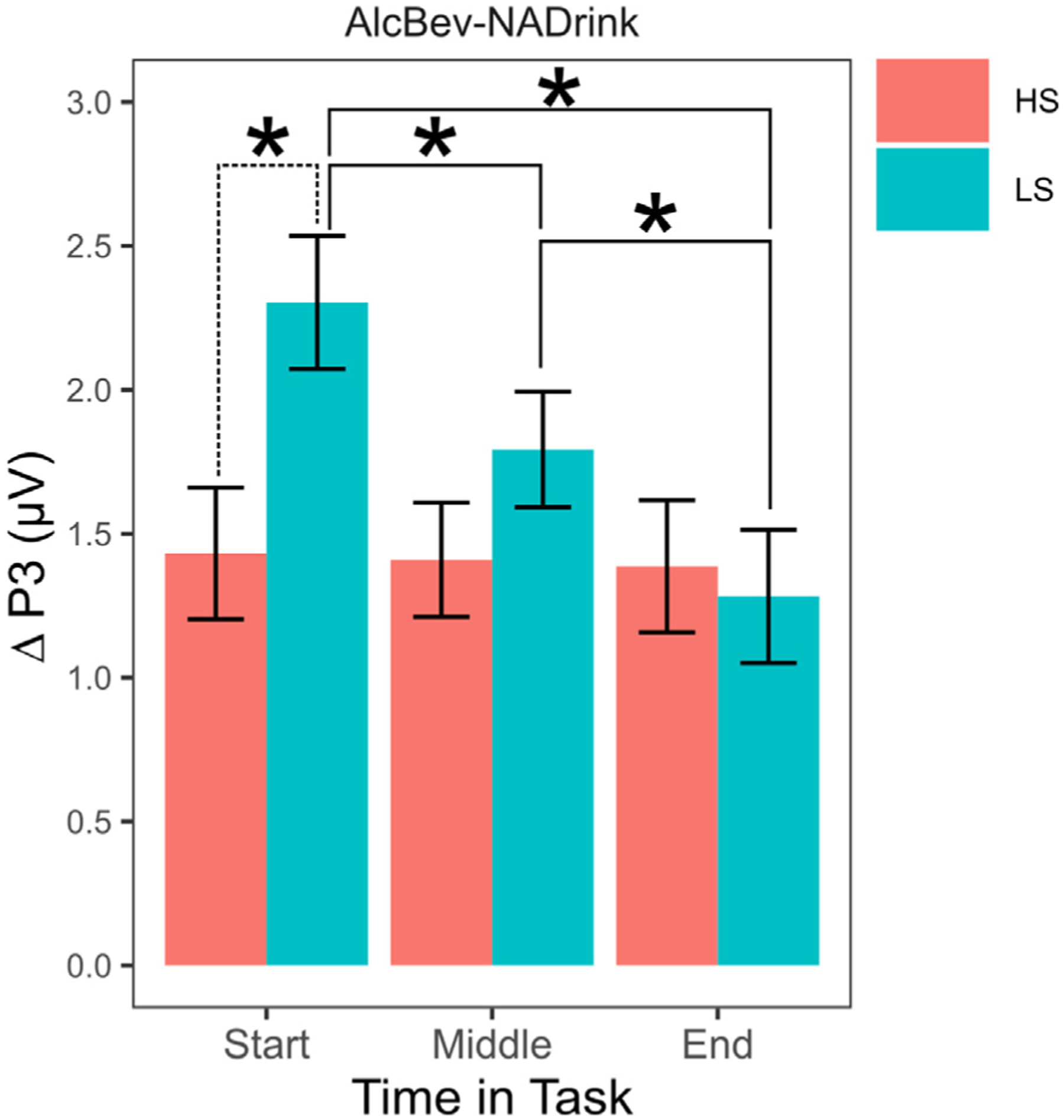
Differences in mean amplitude (μV) of P3 responses to alcohol and nonalcohol picture stimuli at different times in the task as a function of alcohol sensitivity. Alcohol = alcohol beverage pictures. NADrink = nonalcohol drink pictures. Start = first artifact-free trial for each person. Middle = artifact-free trial that bisects each person’s set of artifact-free trials. End = final artifact-free trial for each person. Covariate-adjusted LMM-estimated *M* P3 difference scores shown at different relative times in the picture viewing task. Error bars = ± 1 *SE*. LMM-estimated *M* ± *SE* were derived twice: once holding zASQ at *z* = −1 SD, which corresponds to high sensitivity (HS) phenotypes, and once holding zASQ at *z* = + 1 SD, which corresponds to low sensitivity (LS) phenotypes. * = *p* < .05. Dashed line = within-timepoint, alcohol sensitivity phenotype comparison. Solid line = within-phenotype, timepoint comparisons.

There was a significant interaction between ASQ scores, image category, and linear time, *F* (2, 943143) = 13.47, *p* < .001, *η*2 = 0.008, but not quadratic time, *F* (2, 943265) = 1.53, *p* = .216, *η*2 = 0.001 (see Table S3 + S4).^8^ Inspection of the within-task trajectories of the P3 response by cue type (see Fig. 2) revealed that there was a sensitization-like within-task trajectory of the alcohol and nonalcohol drink cue-elicited P3, and that the alcohol cue-elicited P3 was elevated in its entirety for LS compared to HS phenotypes. The P3 response to the nonalcohol drink cue was initially similar between LS and HS phenotypes and grew increasingly divergent across the task. Additionally, for LS phenotypes, the within-person, addiction-specific cue reactivity (ACR), captured by differences between alcohol and nonalcohol beverage cue-elicited P3 responses, was largest at the start of the task and decreased as the task progressed, whereas for HS phenotypes, the difference was unchanged across the task (Fig. 3). ACR was significantly larger for LS than HS phenotypes across the first quarter of the task, with the maximal phenotypic difference at the start of the task (as shown in Fig. 3). ACR remained numerically larger for LS compared to HS phenotypes across the second quarter of the task, but became numerically equivalent by the middle of the task (as shown in Fig. 3). In contrast, the within-person oddball (response target) effect (OE), captured by differences between beverage and non-beverage neutral cue-elicited P3 responses, exhibited a similar pattern for LS and HS phenotypes: smallest at the start of and increasing across the task. However, by the end of the task, the OE was significantly larger for LS compared to HS phenotypes (Fig. S5).

## 4. Discussion

### 4.1. Within-session trajectory of reward-related vs. neutral cue-elicited P3/LPP responses

Hypothesis 1 was partially supported. The P3 response to the standard/non-target stimuli (low-arousal, neutral-valence object/scene pictures) showed a habituation-like pattern, but P3 response to the oddball/target stimuli (alcohol beverage and nonalcohol drink pictures) did not. The latter *grew* in magnitude across the picture viewing task (until reaching an apparent asymptote)–a sensitization-like trajectory (see Figs. 2, S2, and S6). Prior work on oddball tasks with simple visual stimuli suggests that habituation of the cognitive P3 response is more readily observed in passive relative to active tasks, and that in active tasks, habituation will occur only when oddball repetition is sufficiently massed in time [32, 34, 68, 73]. Prior work on affective picture viewing tasks suggests that such conditions will result in habituation of the LPP response to different pictures, but not habituation of the affective significance effect (viz., the LPP to affectively valenced pictures continues to be larger than the LPP to affectively neutral pictures even when the LPP response to each picture type itself exhibits habituation across repeated elicitation) [10, 13, 22]. Thus, sufficiently massed repetition in time may be able to induce habituation of the attentional orienting response (OR) components of the P3/LPP [68], but not its affective or associative response components [22].

The oddball picture viewing task in the current study involves massively repeated presentation of the affectively neutral cues (non-target/standard stimuli), but a relatively limited number of beverage cue presentations (response target/oddball stimuli). It is thus not surprising that the P3/LPP response to the affectively neutral cues showed a habituation-like trajectory across trials, as expected based on the literature. Nonetheless, in the absence of massed repetition, the P3/LPP literature would predict that the P3/LPP response to the beverage cues (oddball/target stimuli) would remain relatively stable across trials in a single session. To the extent that the picture viewing task is *de facto* extinction (non-reinforcement) of naturally conditioned cues for ingested rewards, such as alcohol and nonalcohol beverages, the learning literature would predict a habituation/extinction-like trajectory across trials for the beverage cue-elicited P3/LPP. Instead, it showed a sensitization-like trajectory.

Although sensitization is rarely observed outside of specific paradigms (e.g., fear-potentiated startle, [35, 49, 56, 82]), the learning literature does describe circumstances under which acute sensitization of stimulus-elicited responses can occur. One such circumstance is the combination of high stimulus intensity and low stimulus presentation frequency (see [36]). Thus, the within-session sensitization of beverage cue-elicited P3 responses might be due to the combination of a low frequency of presentation and the higher intensity of the beverage cues relative to neutral cues. To the extent that low-frequency, repeated, non-reinforced exposure to visual alcohol cues is experienced in daily life, acute sensitization of covert attention to, and/or affective/motivational processing of, those cues (as indexed by the P3/LPP response) could contribute to the intrusion of alcohol use-related thoughts or emergence of conscious craving (desire) for alcohol (see [44]).

Finally, we and others also have argued that the addiction-relevant component of variation in P3/LPP response to alcohol/drug reward cues is best isolated by taking into account the person’s P3/LPP response to nondrug reward cues [55, 84]. If so, then it is enhanced reactivity to alcohol reward cues *relative to* other reward cues that indexes risk for problematic substance use related to incentive salience over-attribution. In the current study, despite sensitization of the P3 response to individual beverage cues across trials, the addiction cue-specific reactivity (ACR), captured in the difference between alcohol and nonalcohol beverage cue-elicited P3 responses, tended to exhibit a habituation/extinction-like trajectory across trials (see Figs. 3, S3, and S7). This may be due to differential ceiling effects on sensitization of alcohol/drug vs. nonalcohol/drug reward cue-elicited P3 responses (see Figs. 2 and S2). Although the functional (i.e., psychological) significance of this within-session trajectory for ACR is unclear, it does provide an explanation for the limited psychometric reliability of commonly used across-trial average ACR scores [16]. Furthermore, it suggests that researchers interested in using such P3/LPP difference scores as a measure of individual differences in incentive salience-based risk for problematic alcohol/drug use should consider estimating within-session trajectories in order to be able to extract the model-estimated difference score at the start of the session (i.e., before habituation/extinction effects and/or before ceiling effects on sensitization). These model-estimated difference scores may have greater psychometric reliability than the commonly used across-trial average difference score.

### 4.2. Moderation of alcohol cue-elicited P3/LPP responses by alcohol sensitivity levels

Hypotheses 2 and 3 were partially supported. Consistently across the task, the P3 response to alcohol cues was larger among individuals with lower alcohol sensitivity phenotypes (LS) compared to individuals with higher alcohol sensitivity (HS) phenotypes, but only significantly so at the midpoint in the task (see Fig. 2). Moreover, the P3 response to alcohol beverage cues *relative to* nonalcohol beverage cues—that is, addiction cue-specific reactivity (ACR)—was significantly larger for LS compared to HS phenotypes, but only significantly so across the first quarter of the task. This differential P3 response, which may capture addiction-related pathology in cue/reward incentive value attribution processes, exhibited within-task habituation/extinction for LS but not HS individuals (see Fig. 3). The P3 response to alcohol and nonalcohol beverage cues *relative to* control nonreward neutral cues was larger for LS compared to HS phenotypes, but only at the end of the task. This differential P3 response, which may capture domain-general top-down (task-based response target) and bottom-up (novelty salience) incentive value attribution processes, exhibited within-task sensitization for LS and HS phenotypes alike (see Fig. S5). Together, these findings provide in principle replication of a prior study by Martins et al. [54] that used an independent sample, a different picture set, and an evaluative categorization task as well as a different EEG recording system. As in the current study, Martins et al. found that alcohol sensitivity levels significantly predicted the overall magnitude of the P3 response to alcohol beverage images but not the overall magnitude of the P3 response to nonalcohol beverage images or neutral images (also see [5, 10]). Given that P3/LPP response magnitude is theorized to index the integrated (extrinsic or top-down + intrinsic or bottom-up) incentive-motivational value of the eliciting stimulus (see [37]), the current findings add to a growing body of evidence that LS drinkers over-attribute incentive-motivational value to alcohol cues, whether naturally conditioned [5, 10, 15, 54] or newly conditioned in the laboratory [25].

Hypothesis 4 was not supported. Both LS and HS drinkers exhibited within-session sensitization of the P3 response to alcohol cues (as well as of the P3 response to nonalcohol reward cues) rather than the expected within-session habituation pattern. Additionally, exploratory simple slopes analysis (see Table S5) indicated that neither the *amount* of growth in P3 response to alcohol cues from trial to trial nor the *rate* of its growth across trials differed significantly between LS and HS phenotypes (see also Fig. 2). Thus, enhanced P3 responses to alcohol cues for LS compared to HS phenotypes do not stem from differential rates or patterns of trial-by-trial adaptation of the P3 response to alcohol cues. This finding suggests that the association between alcohol sensitivity levels and heightened P3 responses to alcohol cues reflects a trait-like difference at the level of relatively stable physiological or psychological processes (e.g., conditioned [learned] affective/biological significance) rather than those that fluctuate more from moment to moment (e.g., craving, hunger, thirst, hormones). Nonetheless, the *amount* of growth in P3 response to nonalcohol reward cues from trial to trial was significantly larger for LS compared to HS phenotypes despite the *rate* of its growth across trials being similar between phenotypes (see Fig. 2, Table S5). Thus, the ability of detect enhanced P3 responses to alcohol cues *relative* to nonalcohol reward cues, that is enhanced ACR, for LS compared to HS phenotypes may be limited by differential ceiling effects on alcohol vs. nonalcohol reward cue sensitization across the task.

### 4.3. Limitations

Despite its strengths, the current study also was limited in ways that bear on the generalizability of its findings. First and foremost, although large in its size and balanced in terms of male and female representation, the sample was highly homogenous in its sociodemo-graphics (age [emerging adults], ethnicity/race [non-Hispanic White], handedness [Right dominant], education [college students], and culture/nationality [U.S.A.]), which may limit generalizability. Additionally, many chronic illnesses and medical conditions were exclusionary, which increases confidence that our findings are not driven by some other biomedical factor confounded with alcohol sensitivity but also may limit their generalizability. Second, the measure of alcohol sensitivity used in the current study (i.e., the Alcohol Sensitivity Questionnaire [ASQ]) differs from the measure used in the majority of studies that have established LS as an AUD risk-conferring endophenotype (i.e., the Self-Rating of the Effects of Alcohol [SRE] scale; [76]). Nonetheless, ASQ and SRE total scores are highly correlated [24], in keeping with the idea that these measures index the same underlying construct. Third, although we had good coverage of relatively heavy alcohol use behavior, the sample consists of individuals relatively early in their history of alcohol involvement (see Table 1), so it remains to be seen whether individuals with heavier and longer histories of alcohol use, especially individuals with histories of AUD treatment involvement, will exhibit similar within-session sensitization of the P3 response to alcohol and nonalcohol reward cues. Finally, although we contextualize individual differences in P3 response to alcohol cues within the broader literature on individual differences in P3/LPP responses to drug cues, it remains to be seen whether P3/LPP responses to cues for nonalcohol drug rewards also will exhibit within-session sensitization. Additionally, given that the control nonalcohol reward cue-elicited P3/LPP response also exhibited within-session sensitization, future studies should explore the trial-by-trial trajectory of P3/LPP responses to cues for other ingested rewards as well as the P3/LPP responses to visual cues for non-ingested rewarding stimuli (e.g., money, positive affective states, socializing, sex). Similarities and differences in the within-session trajectory of P3/LPP responses to different classes of rewarding stimuli (e.g., ingestion-related vs. non-ingested) can inform theories of the P3/LPP component as an integrative neural index of stimulus incentive-motivational value.

## 5. Conclusion

Acute sensitization of incentive value attribution to alcohol cues, as indexed by within-task sensitization of the P3/LPP response to alcohol cues, may be a mechanism for alcohol cue-triggered attentional biases in the absence of immediate reward receipt for individuals across the alcohol sensitivity spectrum, but individuals reporting lower sensitivity to alcohol were found to more consistently over-attribute incentive value to alcohol cues. Additionally, the heightened P3/LPP response to alcohol cues among individuals reporting lower compared to higher alcohol sensitivity appears to reflect a trait-like difference in the process of incentive salience attribution to alcohol cues rather than differential sensitization or habituation/extinction of reactivity in the face of repeated non-reinforced cue exposure within a single episode. Identifying the neurobiological underpinnings of this trait-like difference in the affective/motivational significance of alcohol cues may clarify the nature of LS-based risk for alcohol use disorders.

## Supplementary Material

Supplemental Information

## Figures and Tables

**Table 1:** Sample characteristics.

	n (%)		Equal representation? X^2,^ df, *p*
Female	159 (55)		3.35, 1, .067
Ethnicity			
Hispanic	22 (8)		204, 1, < .001
Race			1008, 5, < .001
American Indian/Alaskan Native	2 (<1)		
Native Hawaiian/Pacific Islander	0 (0)		
Asian	11 (4)		
Black	10 (5)		
White	248 (86)		
Multiple Selected	14 (5)		
None Selected	2 (<1)		
Handedness^1^			
Right-Handed	257 (90)		179, 1, < .001
	Female	Male	
	M (SD)	M (SD)	Equal between sexes? *U, p*
Age, yr	19.58 (0.74)	19.49 (0.72)	9621, .428
Height, m	1.67 (0.06)	1.81 (0.07)	18776, < .001
Weight, kg	68.02 (15.08)	80.17 (17.09)	15185, < .001
BMI, kg/m^2^	24.39 (5.49)	24.50 (4.85)	10714, .387
Alcohol Use-Related Characteristics			
Age at First Alc. Intox., yr	16.59 (1.35)	16.59 (1.54)	9716, .771
Age at Reg. Alc. Use, yr	17.44 (1.15)	17.20 (1.26)	8764, .158
Years Since First Alc. Intox.	3.03 (1.41)	2.89 (1.55)	8980, .412
Years Since Reg. Alc. Use	2.15 (1.12)	2.29 (1.27)	10158, .471
Past Year Alcohol Use			
Drinking days per week	1.79 (1.35)	1.87 (1.45)	10383, .691
Drinks per drinking day	4.75 (3.15)	6.57 (3.46)	13828, < .001
AlcQF^2^	9.50 (10.38)	13.07 (14.16)	11794, .015
Max drinks in 24 hr	9.31 (4.64)	15.23 (7.34)	15236, < .001
Binges per week	0.86 (0.92)	1.21 (1.11)	12360, < .001
Raw ASQ score^3^	4.10 (1.47)	6.27 (1.95)	13707, < .001
Standardized ASQ score	−0.02 (0.76)	−0.01 (0.74)	8536, *p* = .669
AUD Symptom Count	2.09 (2.08)	2.44 (2.45)	10798, .365
	n (%)	n (%)	Equal between sexes? X^2,^ df, *p*
AUD Category			2.34, 3, .505
No AUD (0–1 symptoms)	77 (48)	59 (46)	
Mild AUD (2–3 symptoms)	43 (27)	30 (23)	
Moderate AUD (4–5 symptoms)	29 (18)	25 (20)	
Severe AUD (6 + symptoms)	10 (6)	14 (11)	

Note. Total N = 287.

AlcQF = past year alcohol quantity-frequency composite score, computed as the product of past year drinking days per week and drinks per drinking day per week.

1 Right-handedness was defined as an Edinburgh Handedness Inventory short-form score of 61 or above [83].

2 Out of 287 participants, 108 (38%) individuals had AlcQF scores ≤ 5, 60 (21%) had scores ≤ 10, and 42 (15%) had scores ≥ 20, which correspond to light, moderate, and heavy alcohol use phenotypes.

3 Out of 287 participants, 97 (37%) had raw ASQ scores ≤ 3, 84 (32%) had ASQ scores between 4–5, and 77 (30%) had scores ≥ 6, which correspond to high, moderate, and low alcohol sensitivity phenotypes.

## Data Availability

Data will be made available on request.

## Footnotes

1 Please note that the theoretical neural sensitization construct invoked here to explain potential within-session growth of the alcohol/drug cue-elicited P3 refers to the acute (i.e., within-session) form of a naturally occurring, non-associative learning process best described by [36]. In contrast, the theoretical *incentive sensitization* construct described in the ISTA [8,72] refers to a chronic (i.e., between-sessions) sensitization process driven by accrual of neurobiological adaptations to repeated drug exposure and occurring in a specific neural system: the neural circuitry responsible for attribution of incentive-motivation significance to stimuli. Nonetheless, it is conceivable that the level of response to an alcohol/drug cue could reflect the expression of both kinds of sensitization effects (in addition to effects of other factors).

2 Data collection for the second and third laboratory sessions of the parent study was ongoing at the time of this report. Aims of the parent study include psychometrics (e.g., internal consistency reliability, long-term test-retest reliability) on different laboratory task-based measurements of alcohol cue reactivity (e.g., alcohol cue-elicited P3 amplitude, alcohol cue-elicited behavioral approach tendency), factor analysis of the different measures, and testing of their predictive utility using ecological momentary assessment (EMA) of alcohol craving and consumption in daily life.

3 Based on our prior psychometric work with this sample and task [15], 20 artifact-free segments yield P3 scores with excellent internal consistency and adequate test-retest stability across 8–10 months.

4 The *M* ± *SD* number of artifact-free epochs per condition was 36 ± 4 for oddball stimuli (alcohol or nonalcohol pictures) and 294 ± 37 for standard stimuli (neutral pictures). Following [53], the standardized measurement error (SME) was ± 0.46 μV for oddball/target stimuli, and ± 0.17 μV for standard/non-target stimuli.

5 Across participants, electrodes, trials, and image types, there were 946,305 observations.

6 For brevity, we present only results using the ASQ total score as an index of alcohol sensitivity. Given that a previous study using a similar task found evidence for enhanced alcohol beverage cue-elicited P3 mean amplitude relative to nonalcohol drink cue-elicited P3 mean amplitude as a function of scores on the “heavy” subscale of the ASQ but not for scores on the “light” subscale of the ASQ [54], we also ran all analyses using either the “light” or “heavy” subscale-specific scores as the index of alcohol sensitivity. Results were unchanged (albeit less statistically significant) and the pattern of results did not differ between subscales.

7 Results were unchanged when testing alcohol sensitivity and alcohol use effects in separate models rather than a joint model, although the magnitude of differences at phenotypic extremes of alcohol sensitivity and alcohol use were somewhat attenuated when estimated in separate models.

8 Other significant effects not relevant to our hypothesis, such as those involving AlcQF scores, are presented in Supplemental Information. Of note, the pattern of AlcQF score effects was dissimilar from the pattern of ASQ score effects. Specifically, although within-session sensitization was still observed in the model-estimated means for AlcQF scores corresponding to lighter and heavier alcohol use patterns alike, heavier compared to lighter alcohol use was associated with slightly *reduced* amplitude P3 responses to alcohol and nonalcohol reward cues. This is consistent with *reduced* amplitude P3 response to oddball (infrequent response-target) compared to standard (frequent non-target) stimuli in the canonical oddball tasks (e.g., the rotated heads task) among individuals with alcohol use disorders [38]. However, this *reduced* oddball P3 phenotype has been demonstrated to index heritable risk for externalizing psychopathology spectrum disorders broadly rather than alcohol use disorder specifically [12, 30, 42, 65]. The slightly *reduced* amplitude P3 response to the nonalcohol reward cue associated with heavier compared to lighter alcohol use also is consistent with *reduced* motivational reactivity to non-drug rewards among individuals with, or at elevated risk for, alcohol/drug use disorders [9, 33, 55, 90].
