# Supplementary material for: Acute sensitization of the P3 event-related potential response to beverage images and the risk for alcohol use disorder
Source: Addict Neurosci. Author manuscript; Available in PMC 2022 Dec 1. (PMC9681121; doi:10.1016/j.addicn.2022.100041)
Supplement: Supplemental Information [file NIHMS1850133-supplement-Supplemental_Information.docx]

**Supplemental Information**

This supplemental material accompanies the article titled, “Acute Sensitization of the P3 Event-Related Potential Response to Beverage Images and the Risk for Alcohol Use Disorder,” by RU Cofresí, TM Piasecki, and BD Bartholow.

**Method**

**Participants**

Participants were screened and enrolled between 08/10/2018 and 03/22/2021, when study enrollment closed. Prospective participants were recruited from the University of Missouri (MU) and Columbia, MO community using multiple channels: postings to MU weekly informational e-newsletter received by students, faculty, and staff; flyers around MU campus; mass screening of undergraduate students enrolled in an large introductory psychology course at MU; flyers around Columbia, MO community (e.g., community college, recreation centers, gyms, music shops and venues, restaurants and pubs); digital ad placement to young adults in Mid Missouri counties (Amplified Digital, St. Louis, MO); word-of-mouth (e.g., website link sharing by prospective and enrolled participants). Participants were invited to the laboratory for session 1 if they were eligible according our inclusion-exclusion criteria. Inclusion criteria were: 18 to 20 years of age at enrollment, ability to read and write English, normal or corrected to normal visual acuity, regular alcohol use (at least monthly alcohol use in the past year and at least 1 binge drinking episode in the past 6 months). Exclusion criteria were: history of neurological disease (e.g., epilepsy); history of prior head injuries that resulted in loss of consciousness for over 2 min; metal plates or implants inside the skull; non-removable metal body jewelry; very sensitive skin; hairstyles that interfere with EEG recording (e.g., beaded or braided hair, bald or clean shaven); history of unsuccessful attempts to quit or moderate alcohol use. Participants were compensated $15 for the eligibility screening survey^[[1]](#footnote-1)^, $13/hr for session 1, and $13.50/hr for session 2.

**Oddball Picture Viewing Task**

All picture stimuli were centered on the screen and presented on an opaque gray background. Picture stimuli subtended 14 horizontal x 15 vertical degrees of visual angle. In all blocks, 80% of trials consisted of non-beverage neutral pictures and 20% of trials consisted of beverage pictures (10% alcohol, 10% nonalcohol). Non-beverage neutral pictures were drawn from the Internal Affective Picture System (IAPS) (Lang et al., 2008). These were images rated as low in arousal and near the scale midpoint in valence.^[[2]](#footnote-2)^ Alcohol and nonalcohol beverage pictures were drawn from the “passive” subset (displaying only the bottle and/or empty/full glass on a bland white background) of the Amsterdam Beverage Picture Set (ABPS) (Pronk et al., 2015),^[[3]](#footnote-3)^ and supplemented with pictures of four alcohol beverages taken by a local professional photographer (based on pretest data indicating favored alcohol drinks among the population from which the sample was drawn; pictures displayed only the beverage on a bland white background as in the ABPS).^[[4]](#footnote-4)^

Participants were instructed to press one of two buttons on a button box whenever a beverage picture was presented (left or right indicating alcohol beverage or nonalcohol beverage; response mapping was counterbalanced across participants), and to withhold responding to non-beverage neutral pictures. Each trial began with a fixation cross (duration varied randomly from 1000 to 2000 ms; average across trials was 1500 ms) followed immediately by a picture stimulus for 1000 ms. During practice trials only, each stimulus was followed by feedback (presented for 1500 ms) on response time and accuracy. No feedback was presented during experimental trials. Participants completed one block of 20 practice trials followed by two blocks of 200 experimental trials with a 1-3 min intervening rest period. Experimental trial block stimulus sequences were generated on-the-fly by random selection without replacement from a list of stimuli (2 cycles of random selection per block).^[[5]](#footnote-5)^

***Electrophysiological Recording and Event-Related Potential (ERP) Component Scoring***

The electroencephalogram (EEG) was recorded from 32 sintered Ag/AgCl ring electrodes embedded in an elastic fabric cap (BrainCap; EASYCAP, LLC, Herrshing, Germany) following the expanded 10-20 placement system (American Electroencephalographic Society, 1991). Plastic syringes (and blunt tip needles when hair was thick) were used to gently abrade the scalp and fill the electrodes with Abralyt HiCl (EASYCAP, LLC). Electrode impedances were kept below 10 kΩ. The EEG was sampled at 512 Hz, referenced to the right mastoid, using a Grael v2 amplifier, and acquired using Curry 8 software (both from Compumedics Neuroscan, LLC, Charlotte, NC). A ground electrode was placed at AFz. The Grael v2 amplifier hardware contains a DC-coupled high-pass filter and applies a 3 dB anti-aliasing low-pass filter online (effective recording bandwidth at 512 Hz sampling rate = 0 to 143 Hz).

After acquisition, each participant’s data underwent a standardized offline pre-processing pipeline implemented in EEGlab (Delorme & Makeig, 2004) and ERPlab (Lopez-Calderon & Luck, 2014). An average mastoid reference was derived, and EEG data were resampled at 256 Hz. DC bias was removed. A 2^nd^ order Butterworth bandpass filter was applied with half-amplitude cut-offs of 0.1 and 30 Hz. Sinusoidal noise (e.g., AC power line fluctuations, fluorescent lighting hum) was attenuated using the CleanLine plug-in for EEGlab (Mullen, 2012). Using session notes and CleanLine, “bad” (e.g., excessively noisy) electrodes were identified and removed, and independent components analysis (ICA) was conducted on continuous EEG data from the remaining electrodes. The ADJUST plug-in for EEGlab (Mognon et al., 2011) was used to identify and remove ICs corresponding to blinks as well as eye movements and other artifacts. On average, 6 ICs were removed per person. After removal of artefactual ICs, previously “bad” electrodes were interpolated using the spherical spline method in EEGlab. On average, 1 electrode was interpolated per person. Next, EEG data at every electrode were segmented into stimulus-locked epochs (-200 to 1000 ms), discarding data from trials in which the picture was incorrectly categorized (≈1%). Finally, moving peak-to-peak thresholds (± 75 µV, window: 400 ms, step: 100 ms) and point-to-point difference thresholds (±20 µV) were applied to identify artefactual voltage deflections at any electrode in the montage of interest (occipitoparietal scalp: O1, O2, PO7, PO8, P7, P8, P3, P4, Pz) for a given epoch. Epochs with artifacts were discarded (≈10%).

The P3 components were identified in the electrode-level grand average ERPs based on previously reported latency and scalp topography (Hajcak & Foti, 2020; Larson et al., 2014; Linden, 2005; Polich, 2007; West et al., 2005). The P3 component manifested here as a slow positive deflection peaking at approximately 500 ms post-stimulus over occipital and parietal locations. Consequently, P3 amplitudes were measured on every retained trial as the average voltage from 300 to 700 ms post-stimulus onset minus the average voltage 200 ms pre-stimulus onset at 9 electrodes over occipitoparietal scalp (O1, O2, PO7, PO8, P7, P8, P3, P4, Pz). Grand average ERP waveforms, obtained by averaging all artifact-free epochs, separately by picture type, are shown in **Figure 1** (main text). Scalp topographies of the grand average ERPs are shown separately by picture type in **Figure S1**.

***Alcohol Sensitivity***

During eligibility screening, participants completed the 15-item Alcohol Sensitivity Questionnaire (ASQ) (O’Neill et al., 2002). The ASQ asks respondents to indicate whether they have ever experienced each of 15 effects from drinking alcohol (e.g., feeling more talkative; feeling dizzy) and, for each effect endorsed, to estimate the minimum number of drinks they can consume before experiencing the effect, or the maximum number of drinks they can consume without experiencing the effect. For current purposes, for each participant the average number of drinks required to experience any given acute effect was computed (ASQ Total score). Given that the number of effects endorsed correlates with the number of drinks reported on each item (see Lee et al., 2015), ASQ Total scores were submitted to standardized person-mean imputation. Specifically, ASQ summary scores were computed as the average of the standardized (i.e., z-score transformed) number of drinks reported for all endorsed effects, such that more positive ASQ z-scores indicate lower alcohol sensitivity. Also, given dramatic sex differences in alcohol sensitivity attributable to differences in alcohol pharmacokinetics between males and females (e.g., Gandhi et al., 2004), ASQ z-scores were stratified by sex to avoid conflating alcohol sensitivity and sex.

***Alcohol Use***

Each subheading below corresponds to an alcohol use measure reported in Table 1 in the main text.

**Drinking days per week.** “During the last 12 months, how often did you usually have any kind of drink containing alcohol?” The response options were as follows, with scaling indicated in parentheses: “Every day” (7); “5 to 6 times a week” (5.5); “3 to 4 times a week” (3.5); “Twice a week” (2); “Once a week” (1); “2 to 3 times a month” (2.5 / 4 = 0.625); “Once a month” (1 / 4 = 0.25); “3 to 11 times in the past year” ( 7 / [4 x 12] = 0.146); “1 or 2 times in the past year” (1.5 / [4 x 12] = 0.03125); “I did not drink alcohol in the last year” (0).

**Drinks per drinking day.** “During the last 12 months, how many alcoholic drinks did you usually have on a typical day when you drink alcohol?” The response options were as follows, with scaling indicated in parentheses: “25 or more drinks” (25); “19 to 24 drinks” (21.5); “16 to 18 drinks” (17); “12 to 15 drinks” (13.5); “9 to 11 drinks” (10); “7 to 8 drinks” (7.5); “5 to 6 drinks” (5.5); “3 to 4 drinks” (3.5); “2 drinks” (2); “1 drink” (1); “I did not drink alcohol in the last 12 months” (0).

**Max drinks in 24 hr.** “During the last 12 months, what is the largest number of drinks containing alcohol that you drank within a 24-hour period?” The response options were as follows, with scaling indicated in parentheses: “36 drinks or more” (36); “24 to 35 drinks” (29.5); “18 to 23 drinks” (20.5); “12 to 17 drinks” (14.5); “8 to 11 drinks” (9.5); “5 to 7 drinks” (6); “4 drinks” (5.5); “3 drinks” (3); “2 drinks” (2); “1 drink” (1); “I did not drink alcohol in the last 12 months” (0).

**Binges per week.** “During the last 6 months, how often did you have 5 or more (for men) or 4 or more (for women) drinks containing any kind of alcohol within a two-hour period? Choose only one response.” The response options were as follows, with scaling indicated in parentheses: “Every day” (7); “5 to 6 days a week” (5.5); “3 to 4 days a week” (3.5); “Twice a week” (2); “Once a week” (1); “2 to 3 days a month” (2.5 / 4 = 0.625); “Once a month” (1 / 4 = 0.25); “3 to 11 days in the past 6 months” (7 / [4 x 6] = 0.292); “1 or 2 days in the past 6 months” (1.5 / [4 x 6] = 0.062); “Not at all” (0).

**Age at first alcohol intoxication.** “How old were you the first time you got drunk, that is, your speech was slurred or you were unsteady on your feet?” Participants were asked to indicate what age in years.

**Age at onset of regular alcohol use.** “At what age did you begin to drink regularly; that is, drinking at least once a month for 6 months or more?” Participants were asked to indicate what age in years.

**Years since first alcohol intoxication relative to age at lab session.** This measure was computed as the difference between each participant’s age and their self-reported age at first alcohol intoxication.

**Years since onset of regular alcohol use relative to age at lab session.** This measure was computed as the difference between each participant’s age and their self-reported age at onset of regular alcohol use.

**Alcohol Use Disorder (AUD) symptom count.** This measure was derived from the Mini International Neuropsychiatric Interview (MINI) AUD module (Sheehan et al., 1998).

**AUD category.** This measure groups participants based on AUD symptom counts according to thresholds in the MINI AUD module.

**Procedure**

Participants were asked to abstain from alcohol for 24 hr and to refrain from eating for 30 min prior to their scheduled laboratory session. Upon arrival, participants were shown to a private room and provided informed consent. Breath alcohol concentration (BrAC) was measured using the Alco-Sensor IV (Intoximeters, St. Louis, MO) in order to confirm sobriety (BrAC = .000 g%). Two participants had to be rescheduled because they arrived with non-zero BrAC. Participants were then prepared for EEG recording as described in (Light et al., 2010). Participants completed the picture viewing task and two other cognitive tasks not reported here. Afterward, EEG recording electrodes were removed and participants were shown to a restroom where they could wash the recording gel out of their hair. Participants then had a 20 min break during which they ate snacks and drank water. After the break, they completed a behavioral task followed by a self-report questionnaire battery and semi-structured clinical interview. Finally, participants were provided instructions on the ecological momentary assessment component of the study and compensation for the session.

**Results**

***Within-task trajectory of cue-elicited P3 responses ignoring individual differences in alcohol use disorder risk-relevant phenotypes***

There were significant interactions of linear and quadratic time with image category (**Table S2**+**S4**). Follow-up simple slopes analysis indicated significant *positive* slope of linear time for beverage cues and non-significant *negative* slope of linear time for neutral cues, and significant *negative* slopes of quadratic time for all cue types (**Table S5**). Thus, the within-task trajectory of the P3 response differed by cue type: a sensitization-like trajectory for beverage cues, and a habituation-like trajectory for neutral cues (**Figure S2**). **Figure S3** shows that the addiction cue-specific reactivity (ACR), captured by the difference between P3 to alcohol and nonalcohol beverage cues, was largest at the start of the task and decreased across the task such that by the end, it was almost halved. In contrast, the oddball (response target) effect (OE), captured by the difference between P3 to beverage and neutral cues, was almost 3 times larger at the middle and end compared to start of the task (**Figure S4**).

Together, these findings indicate that there is within-task sensization of the P3 response to alcohol and nonalcohol reward cues, but rapid habituation of the P3 response to control non-reward cues. The ACR appeared to habituate/extinguish across trials in the task. This may be due to differential ceiling effects on sensitization of the P3 response to alcohol vs. nonalcohol reward cues. In contrast, the OE appeared to sensitize across trials in the task, reaching its asymptotic level by the middle of the task. The different within-task trajectories of the ACR and the OE are consistent with the idea that these two derived (difference score) measures index different constructs.

***Within-task trajectory of cue-elicited P3 responses: exploring the role of individual differences in alcohol use***

Significant interactions between AlcQF (alcohol use) scores, image category, and linear time as well as quadratic time also were detected within the alcohol sensitivity hypothesis-testing model (**Table S3**+**S4**). As in the main text, the model was used to estimate covariate-adjusted means across the task while holding AlcQF scores at the lower quartile, which captures lighter alcohol use (LA) phenotypes, and while holding AlcQF scores at the upper quartile, which captures heavier alcohol use (HA) phenotypes. **Figure S6** shows that alcohol use moderated the within-task trajectories of P3 responses to beverage but not neutral cues. Specifically, there was a tendency for smaller P3 responses to either beverage cue type for the HA compared to LA phenotype. For both LA and HA phenotype, addiction cue-specific reactivity (ACR), captured by the difference between P3 to alcohol and nonalcohol beverage cues, was largest at the start of the task and decreased across it (**Figure S6**). In contrast, the OE was smallest at the start of the task and increased across the task such that, by the middle and end, it was 2-3 times its initial size (**Figure S6**). Compared to the LA phenotype, the HA phenotype exhibited significantly smaller P3 response to alcohol cues (**Figure S7**), and significantly smaller responses to alcohol cues relative to either the nonalcohol drink or neutral cues, but only at the middle of the task (**Figure S7**).

Exploratory simple slopes analysis (**Table S5**) revealed: (i) that the amount of growth in nonalcohol reward cue-elicited P3 response amplitude from trial to trial was significantly greater for the LA compared to HA phenotype; (ii) that trial-by-trial growth in the alcohol and nonalcohol reward cue-elicited P3 response amplitude decelerated similarly for the LA phenotype whereas for the HA phenotype trial-by-trial growth in the nonalcohol reward cue-elicited P3 response amplitude decelerated more quickly than trial-by-trial growth in the alcohol reward cue-elicited P3 response amplitude; and (iii) the trial-by-trial growth in the alcohol reward cue-elicited P3 response amplitude decelerated more quickly for the LA compared to HA phenotype.

Together, these LA and HA phenotype differences are consistent with reduced amplitude P3 response to oddball (infrequent response-target) compared to standard (frequent non-target) stimuli in the canonical oddball tasks (e.g., the rotated heads task) among individuals with alcohol use disorders (Hamidovic & Wang, 2019). Importantly, this "reduced P3" phenotype has been demonstrated to index heritable risk for externalizing psychopathology spectrum disorders broadly rather than alcohol use disorder specifically (Carlson et al., 2007; Gao & Raine, 2009; Iacono et al., 2003; Patrick et al., 2006). Additionally, the LA and HA phenotype differences with respect to the nonalcohol reward cue-elicited P3 specifically are broadly consistent with the idea of reduced motivational reactivity to non-drug rewards among individuals with, or at elevated risk for, alcohol/drug use disorders (Blum et al., 1996; Goldstein et al., 2007; Martins et al., 2021; Zilverstand et al., 2018).

**References**

American Electroencephalographic Society. (1991). American Electroencephalographic Society guidelines for standard electrode position nomenclature. *Journal of Clinical Neurophysiology*, *8*(2), 200–202.

Bartholow, B. D., Henry, E. A., & Lust, S. A. (2007). Effects of Alcohol Sensitivity on P3 Event-Related Potential Reactivity to Alcohol Cues. *Psychology of Addictive Behaviors*, *21*(4), 555–563. https://doi.org/10.1037/0893-164X.21.4.555

Bartholow, B. D., Loersch, C., Ito, T. A., Levsen, M. P., Volpert-Esmond, H. I., Fleming, K. A., Bolls, P., & Carter, B. K. (2018). University-Affiliated Alcohol Marketing Enhances the Incentive Salience of Alcohol Cues. *Psychological Science*, *29*(1), 83–94. https://doi.org/10.1177/0956797617731367

Bartholow, B. D., Lust, S. A., & Tragesser, S. L. (2010). Specificity of P3 Event-related potential reactivity to alcohol cues in individuals low in alcohol sensitivity. *Psychology of Addictive Behaviors*, *24*(2), 220–228. https://doi.org/10.1037/a0017705

Blum, K., Cull, J. G., Braverman, E. R., & Comings, D. E. (1996). Reward Deficiency Syndrome. *American Scientist*, *84*(2), 132–145.

Carlson, S. R., McLarnon, M. E., & Iacono, W. G. (2007). P300 Amplitude, Externalizing Psychopathology, and Earlier- Versus Later-Onset Substance-Use Disorder. *Journal of Abnormal Psychology*, *116*(3), 565–577. https://doi.org/10.1037/0021-843X.116.3.565

Delorme, A., & Makeig, S. (2004). EEGLAB: An open source toolbox for analysis of single-trial EEG dynamics including independent component analysis. *Journal of Neuroscience Methods*, *134*(1), 9–21. https://doi.org/10.1016/j.jneumeth.2003.10.009

Gandhi, M., Aweeka, F., Greenblatt, R. M., & Blaschke, T. F. (2004). Sex differences in pharmacokinetics and pharmacodynamics. *Annual Review of Pharmacology and Toxicology*, *44*(1), 499–523. https://doi.org/10.1146/annurev.pharmtox.44.101802.121453

Gao, Y., & Raine, A. (2009). P3 event-related potential impairments in antisocial and psychopathic individuals: A meta-analysis. *Biological Psychology*, *82*(3), 199–210. https://doi.org/10.1016/j.biopsycho.2009.06.006

Goldstein, R. Z., Tomasi, D., Rajaram, S., Cottone, L. A., Zhang, L., Maloney, T., Telang, F., Alia-Klein, N., & Volkow, N. D. (2007). Role of the anterior cingulate and medial orbitofrontal cortex in processing drug cues in cocaine addiction. *Neuroscience*, *144*(4), 1153–1159. https://doi.org/10.1016/j.neuroscience.2006.11.024

Hajcak, G., & Foti, D. (2020). Significance?... Significance! Empirical, methodological, and theoretical connections between the late positive potential and P300 as neural responses to stimulus significance: An integrative review. *Psychophysiology*, *57*(7), 1–15. https://doi.org/10.1111/psyp.13570

Hamidovic, A., & Wang, Y. (2019). The P300 in alcohol use disorder: A meta-analysis and meta-regression. *Progress in Neuro-Psychopharmacology and Biological Psychiatry*, *95*(July), 109716. https://doi.org/10.1016/j.pnpbp.2019.109716

Iacono, W. G., Malone, S. M., & McGue, M. (2003). Substance use disorders, externalizing psychopathology, and P300 event-related potential amplitude. *International Journal of Psychophysiology*, *48*(2), 147–178. https://doi.org/10.1016/S0167-8760(03)00052-7

Lang, P. J., Bradley, M. M., & Cuthbert, B. N. (2008). *International affective picture system (IAPS): Affective ratings of pictures and instruction manual. Technical Report A-8.*

Larson, M. J., Clayson, P. E., & Clawson, A. (2014). Making sense of all the conflict: A theoretical review and critique of conflict-related ERPs. *International Journal of Psychophysiology*, *93*(3), 283–297. https://doi.org/10.1016/j.ijpsycho.2014.06.007

Lee, M. R., Bartholow, B. D., McCarthy, D. M., Pedersen, S. L., & Sher, K. J. (2015). Two alternative approaches to conventional person-mean imputation scoring of the self-rating of the effects of alcohol scale (SRE). *Psychology of Addictive Behaviors*, *29*(1), 231–236. https://doi.org/10.1037/adb0000015

Light, G. A., Williams, L. E., Minow, F., Sprock, J., Rissling, A., Sharp, R., Swerdlow, N. R., & Braff, D. L. (2010). Electroencephalography (EEG) and event-related potentials (ERPs) with human participants. *Current Protocols in Neuroscience*, *SUPPL. 52*, 1–24. https://doi.org/10.1002/0471142301.ns0625s52

Linden, D. E. J. (2005). The P300: Where in the brain is it produced and what does it tell us? *Neuroscientist*, *11*(6), 563–576. https://doi.org/10.1177/1073858405280524

Lopez-Calderon, J., & Luck, S. J. (2014). ERPLAB: An open-source toolbox for the analysis of event-related potentials. *Frontiers in Human Neuroscience*, *8*(1 APR), 1–14. https://doi.org/10.3389/fnhum.2014.00213

Martins, J. S., Bartholow, B. D., Lynne Cooper, M., Irvin, K. M., & Piasecki, T. M. (2019). Interactive Effects of Naturalistic Drinking Context and Alcohol Sensitivity on Neural Alcohol Cue-Reactivity Responses. *Alcoholism: Clinical and Experimental Research*, *43*(8), 1777–1789. https://doi.org/10.1111/acer.14134

Martins, J. S., Joyner, K. J., McCarthy, D. M., Morris, D. H., Patrick, C. J., & Bartholow, B. D. (2021). Differential brain responses to alcohol‐related and natural rewards are associated with alcohol use and problems: Evidence for reward dysregulation. *Addiction Biology*. https://doi.org/10.1111/adb.13118

Mognon, A., Jovicich, J., Bruzzone, L., & Buiatti, M. (2011). ADJUST: An automatic EEG artifact detector based on the joint use of spatial and temporal features. *Psychophysiology*, *48*(2), 229–240. https://doi.org/10.1111/j.1469-8986.2010.01061.x

Mullen, T. (2012). *CleanLine*. NeuroImaging Tools & Resources Collaboratory. https://www.nitrc.org/projects/cleanline/

O’Neill, S. E., Sher, K. J., & Bartholow, B. D. (2002). Alcohol susceptibility and tolerance in young adults. *Alcoholism: Clinical and Experimental Research*, *26*, 119A.

Patrick, C. J., Bernat, E. M., Malone, S. M., Iacono, W. G., Krueger, R. F., & McGue, M. (2006). P300 amplitude as an indicator of externalizing in adolescent males. *Psychophysiology*, *43*(1), 84–92. https://doi.org/10.1111/j.1469-8986.2006.00376.x

Polich, J. (2007). Updating P300: An integrative theory of P3a and P3b. *Clinical Neurophysiology*, *118*(10), 2128–2148. https://doi.org/10.1016/j.clinph.2007.04.019

Pronk, T., van Deursen, D. S., Beraha, E. M., Larsen, H., & Wiers, R. W. (2015). Validation of the Amsterdam Beverage Picture Set: a controlled picture set for cognitive bias measurement and modification Paradigms. *Alcoholism: Clinical and Experimental Research*, *39*(10), 2047–2055. https://doi.org/10.1111/acer.12853

Satterthwaite, F. E. (1941). Synthesis of variance. *Psychometrika*, *6*(5), 309–316. https://doi.org/10.1007/BF02288586

Sheehan, D. V, Lecrubier, Y., Sheehan, K. H., Amorim, P., Janavs, J., Weiller, E., Hergueta, T., Baker, R., & Dunbar, G. C. (1998). The Mini-International Neuropsychiatric Interview (M.I.N.I): The development and validation of a structured diagnostic psychiatric interview for DSM-IV and ICD-10. In *The Journal of Clinical Psychiatry* (Vol. 59, Issue Suppl 20, pp. 22–33). Physicians Postgraduate Press.

West, R., Jakubek, K., Wymbs, N., Perry, M., & Moore, K. (2005). Neural correlates of conflict processing. *Experimental Brain Research*, *167*(1), 38–48. https://doi.org/10.1007/s00221-005-2366-y

Zilverstand, A., Huang, A. S., Alia-Klein, N., & Goldstein, R. Z. (2018). Neuroimaging Impaired Response Inhibition and Salience Attribution in Human Drug Addiction: A Systematic Review. *Neuron*, *98*(5), 886–903. https://doi.org/10.1016/j.neuron.2018.03.048

| **Table S1** *Associations among alcohol use variables controlling for sex* | | | | | | | | |
| --- | --- | --- | --- | --- | --- | --- | --- | --- |
|  | 1. | 2. | 3. | 4. | 5. | 6. | 7. | 8. |
| 1. ASQ | *-* |  |  |  |  |  |  |  |
| 1. Drinking days per week | .326*** | - |  |  |  |  |  |  |
| 1. Drinks per drinking day | .429*** | .200** | - |  |  |  |  |  |
| 1. AlcQF | .467*** | .722*** | .721*** | - |  |  |  |  |
| 1. Max drinks in 24 hr | .579*** | .459*** | .440*** | .541*** | - |  |  |  |
| 1. Binges per week | .447*** | .675*** | .458*** | .741*** | .490*** | - |  |  |
| 1. Years since first intox. | .071 | .283*** | .071 | .193** | .233*** | .229** | - |  |
| 1. Years since reg. use | .040 | .188*** | .009 | .091 | .211*** | .128* | .639*** | - |
| 1. AUD symptom count | .245** | .395*** | .243*** | .376*** | .443*** | .388*** | .402*** | .242*** |
| *Note*. Entries in column “1.” represent semipartial Pearson correlation coefficients between standardized ASQ scores and alcohol use measures controlling for any potential effect of biological sex in the alcohol use measure. Entries in columns “2.”, “3.”, “4.”, “5.”, “6.”, and “7.” represent partial Pearson correlation coefficients between the two alcohol use measures controlling for any potential effect of biological sex in both measures. Data represent *N* = 287 participants.  * *p < .05*  ** *p < .01*  *** *p < .001* | | | | | | | | |

| **Table S2**  *Parameter Estimates in the Base Model of P3 Mean Amplitude (µV)* | | | | | |
| --- | --- | --- | --- | --- | --- |
| *Fixed Effects* | *b* | *SE* | *t* | *df* | *p* |
| (Intercept) | 4.365 | 0.239 | 18.281 | 290.476 | < .001 |
| Age | -0.008 | 0.199 | -0.041 | 286.884 | 0.968 |
| Sex | -0.084 | 0.147 | -0.57 | 286.851 | 0.569 |
| Handedness | -0.624 | 0.239 | -2.61 | 286.813 | 0.010 |
| Linear Time | -0.061 | 0.061 | -1.006 | 293.565 | 0.315 |
| Quadratic Time | 0.372 | 0.088 | 4.234 | 299.31 | < .001 |
| Alcohol Cue | 7.018 | 0.22 | 31.959 | 299.026 | < .001 |
| NADrink Cue | 5.459 | 0.214 | 25.466 | 299.362 | < .001 |
| Linear Time x Alcohol Cue | 1.266 | 0.048 | 26.471 | 943122.698 | < .001 |
| Linear Time x NADrink Cue | 1.533 | 0.049 | 31.435 | 943079.451 | < .001 |
| Quadratic Time x Alcohol Cue | -1.543 | 0.093 | -16.529 | 943043.013 | < .001 |
| Quadratic Time x NADrink Cue | -1.663 | 0.095 | -17.563 | 942850.515 | < .001 |
| *Random Effects* | | | | | |
| Residual error *SD* | | 7.95 | | | |
| Random intercept *SD* | |  | | | |
|  | | 2.42 _person : electrode_ | | | |
|  | | 2.35 _person_ | | | |
| Random slope *SD*s | |  | | | |
|  | | 0.99 _Linear Time \| person_ | | | |
|  | | 1.39 _Quadratic Time \| person_ | | | |
|  | | 3.65 _Alcohol Cue \| person_ | | | |
|  | | 3.56 _NADrink Cue \| person_ | | | |
| Random intercept-slope correlations | |  | | | |
|  | | -0.07 _Linear Time \| person_ | | | |
|  | | -0.12 _Quadratic Time \| person_ | | | |
|  | | -0.03 _Alcohol Cue \| person_ | | | |
|  | | -0.03 _NADrink Cue \| person_ | | | |
| Random slope-slope correlations | |  | | | |
|  | | -0.15 _Linear Time x Quadratic Time \| person_ | | | |
|  | | 0.01 _Linear Time x Alcohol Cue \| person_ | | | |
|  | | -0.09 _Linear Time x NADrink Cue \| person_ | | | |
|  | | 0.02 _Quadratic Time x Alcohol Cue \| person_ | | | |
|  | | 0.02 _Quadratic Time x NADrink Cue \| person_ | | | |
|  | | 0.88 _Alcohol Cue x NADrink Cue \| person_ | | | |
| Intraclass correlations | |  | | | |
|  | | 0.08 _person : electrode_ | | | |
|  | | 0.07 _person_ | | | |
| *n* | |  | | | |
|  | | 2583 _person : electrode_ | | | |
|  | | 287 _person_ | | | |
| Observations | | 946305 | | | |
| Fixed Effects *R*^2^ / Total *R*^2^ | | 0.07 / 0.24 | | | |
| *Note.*  Image type was represented using two dummy coded variables: Alcohol Cue (1/0) and NADrink Cue (1/0). Neutral Cue served as the reference category (i.e., when Alcohol Cue = 0 and NADrink Cue = 0).  Age is a grand-mean centered covariate representing each person's age in years.  Sex is the effect-coded variable indicating that the person is male (-1) or female (1).  Handedness is the effect-coded variable indicating that the person is right-hand dominant (-1) or not (e.g., ambidextrous, left-handed) (1).  Linear Time is a person-centered covariate representing trial (i.e., observation) number relative to the total number of experimental trials in the task, rescaled such that -1 is the start of the task, 0 is the midpoint, and +1 is the end of the task.  Quadratic Time is the square of Linear Time.  Degrees of freedom (df) for parameter *t*-tests were estimated using Satterthwaite's method (1941). | | | | | |

| **Table S3**  *Parameter Estimates in the Hypothesis-Testing Model of P3 Mean Amplitude (µV)* | | | | | |
| --- | --- | --- | --- | --- | --- |
| *Fixed Effects* | *b* | *SE* | *t* | *df* | *p* |
| (Intercept) | 4.348 | 0.239 | 18.217 | 290.356 | < 0.001 |
| Age | -0.001 | 0.2 | -0.003 | 286.874 | 0.997 |
| Sex | -0.078 | 0.149 | -0.525 | 286.852 | 0.600 |
| Handedness | -0.646 | 0.239 | -2.698 | 286.807 | 0.007 |
| Linear Time | -0.061 | 0.06 | -1.01 | 293.556 | 0.314 |
| Quadratic Time | 0.372 | 0.088 | 4.23 | 299.259 | < 0.001 |
| Alcohol Cue | 7.027 | 0.218 | 32.283 | 299.216 | < 0.001 |
| NADrink Cue | 5.463 | 0.213 | 25.594 | 300 | < 0.001 |
| Linear Time x Alcohol Cue | 1.266 | 0.048 | 26.479 | 943122.433 | < 0.001 |
| Linear Time x NADrink Cue | 1.533 | 0.049 | 31.435 | 943074.218 | < 0.001 |
| Quadratic Time x Alcohol Cue | -1.55 | 0.093 | -16.596 | 943043.697 | < 0.001 |
| Quadratic Time x NADrink Cue | -1.663 | 0.095 | -17.556 | 942848.547 | < 0.001 |
| zASQtotal | 0.214 | 0.221 | 0.971 | 287.32 | 0.332 |
| AlcQF | 0.005 | 0.014 | 0.388 | 287.697 | 0.698 |
| zASQtotal x Alcohol Cue | 0.603 | 0.324 | 1.862 | 299.088 | 0.064 |
| zASQtotal x NADrink Cue | 0.303 | 0.318 | 0.955 | 300.017 | 0.34 |
| AlcQF x Alcohol Cue | -0.057 | 0.02 | -2.868 | 299.124 | 0.004 |
| AlcQF x NADrink Cue | -0.031 | 0.02 | -1.583 | 300.567 | 0.114 |
| zASQtotal x Linear Time | -0.12 | 0.09 | -1.336 | 292.639 | 0.183 |
| AlcQF x Linear Time | -0.006 | 0.006 | -1.047 | 293.966 | 0.296 |
| zASQtotal x Quadratic Time | -0.009 | 0.131 | -0.067 | 297.77 | 0.947 |
| AlcQF x Quadratic Time | -0.003 | 0.008 | -0.338 | 300.008 | 0.735 |
| zASQtotal x Linear Time x Alcohol Cue | 0.121 | 0.071 | 1.716 | 943072.75 | 0.086 |
| zASQtotal x Linear Time x NADrink Cue | 0.365 | 0.072 | 5.055 | 943043.444 | < 0.001 |
| AlcQF x Linear Time x Alcohol Cue | -0.002 | 0.004 | -0.464 | 942948.375 | 0.643 |
| AlcQF x Linear Time x NADrink Cue | -0.013 | 0.004 | -3.014 | 943130.396 | 0.003 |
| zASQtotal x Quadratic Time x Alcohol Cue | -0.147 | 0.137 | -1.069 | 943028.657 | 0.285 |
| zASQtotal x Quadratic Time x NADrink Cue | 0.179 | 0.142 | 1.265 | 943118.162 | 0.206 |
| AlcQF x Quadratic Time x Alcohol Cue | 0.045 | 0.009 | 5.273 | 942968.487 | < 0.001 |
| AlcQF x Quadratic Time x NADrink Cue | 0.005 | 0.009 | 0.542 | 943029.058 | 0.588 |
| *Random Effects* | | | | | |
| Residual error *SD* | | 7.95 | | | |
| Random intercept *SD* | |  | | | |
|  | | 2.42 _person : electrode_ | | | |
|  | | 2.35 _person_ | | | |
| Random slope *SD*s | |  | | | |
|  | | 0.99 _Linear Time \| person_ | | | |
|  | | 1.39 _Quadratic Time \| person_ | | | |
|  | | 3.65 _Alcohol Cue \| person_ | | | |
|  | | 3.56 _NADrink Cue \| person_ | | | |
| Random intercept-slope correlations | |  | | | |
|  | | -0.07 _Linear Time \| person_ | | | |
|  | | -0.13 _Quadratic Time \| person_ | | | |
|  | | -0.04 _Alcohol Cue \| person_ | | | |
|  | | -0.03 _NADrink Cue \| person_ | | | |
| Random slope-slope correlations | |  | | | |
|  | | -0.15 _Linear Time x Quadratic Time \| person_ | | | |
|  | | 0.00 _Linear Time x Alcohol Cue \| person_ | | | |
|  | | -0.09 _Linear Time x NADrink Cue \| person_ | | | |
|  | | 0.03 _Quadratic Time x Alcohol Cue \| person_ | | | |
|  | | 0.02 _Quadratic Time x NADrink Cue \| person_ | | | |
|  | | 0.88 _Alcohol Cue x NADrink Cue \| person_ | | | |
| Intraclass correlations | |  | | | |
|  | | 0.08 _person : electrode_ | | | |
|  | | 0.07 _person_ | | | |
| *n* | |  | | | |
|  | | 2583 _person : electrode_ | | | |
|  | | 287 _person_ | | | |
| Observations | | 946305 | | | |
| Fixed Effects *R*^2^ / Total *R*^2^ | | 0.07 / 0.24 | | | |
| *Note.*  Image type was represented using two dummy coded variables: Alcohol Cue (1/0) and NADrink Cue (1/0). Neutral Cue served as the reference category (i.e., when Alcohol Cue = 0 and NADrink Cue = 0).  Age is a grand-mean centered covariate representing each person's age in years.  Sex is the effect-coded variable indicating that the person is male (-1) or female (1).  Handedness is the effect-coded variable indicating that the person is right-hand dominant (-1) or not (e.g., ambidextrous, left-handed) (1).  Biological Sex is the effect-coded variable indicating that the person is male (-1) or female (1).  Handedness is the effect-coded variable indicating that the person is right-hand dominant (-1) or not (e.g., ambidextrous, left-handed) (1).  Linear Time is a person-centered covariate representing trial (i.e., observation) number relative to the total number of experimental trials in the task, rescaled such that -1 is the start of the task, 0 is the midpoint, and +1 is the end of the task.  Quadratic Time is the square of Linear Time.  AlcQF is a grand-mean centered covariate representing each person’s past year alcohol use quantity-frequency (Q-F) product score.  zASQ-T is a grand-mean centered covariate representing each person’s sex-stratified Alcohol Sensitivity Questionnaire (ASQ) score computed using the standardized person mean imputation method (Lee et al., 2015).  Degrees of freedom (df) for parameter *t*-tests were estimated using Satterthwaite's method (1941). | | | | | |

| **Table S4**  *Tests of Effects in Multi-Level Models of P3 Mean Amplitude* | | | | |
| --- | --- | --- | --- | --- |
| Effects | *F* | *df* | *p* | η^2^ |
| ***Base Model*** | | | | |
| Age | 0.00 | 1,287 | **.968** | 0.000 |
| Sex | 0.32 | 1,287 | **.569** | 0.000 |
| Handedness | 6.81 | 1,287 | **.009** | 0.002 |
| Linear Time | 193.92 | 1,333 | **<.001** | 0.058 |
| Quadratic Time | 56.12 | 1,373 | **<.001** | 0.017 |
| Image Type | 515.73 | 2,327 | **<.001** | 0.309 |
| Image Type x Linear Time | 763.14 | 2, 943186 | **<.001** | 0.457 |
| Image Type x Quadratic Time | 262.59 | 2, 943276 | **<.001** | 0.157 |
| ***Hypothesis-Testing Model*** | | | | |
| Age | 0.00 | 1,287 | .997 | 0.000 |
| Sex | 0.28 | 1,287 | .600 | 0.000 |
| Handedness | 7.28 | 1,287 | **.007** | 0.002 |
| Linear Time | 196.76 | 1,333 | **<.001** | 0.057 |
| Quadratic Time | 56.40 | 1,373 | **<.001** | 0.016 |
| Image Type | 526.77 | 2, 328 | **<.001** | 0.304 |
| Image Type x Linear Time | 763.33 | 2, 943185 | **<.001** | 0.441 |
| Image Type x Quadratic Time | 263.49 | 2, 943274 | **<.001** | 0.152 |
| zASQ | 3.04 | 1,286 | .082 | 0.001 |
| AlcQF | 1.73 | 1,289 | .189 | 0.000 |
| Image Type x zASQ | 2.43 | 2,328 | .090 | 0.001 |
| Image Type x AlcQF | 5.40 | 2,329 | **.005** | 0.003 |
| Linear Time x zASQ | 0.21 | 1,332 | .646 | 0.000 |
| Linear Time x AlcQF | 3.68 | 1,333 | .056 | 0.001 |
| Quadratic Time x zASQ | 0.00 | 1,372 | .987 | 0.000 |
| Quadratic Time x AlcQF | 2.62 | 1,373 | .106 | 0.001 |
| Image Type x Linear Time x zASQ | 13.47 | 2, 943143 | **<.001** | 0.008 |
| Image Type x Linear Time x AlcQF | 4.55 | 2, 943127 | **.011** | 0.003 |
| Image Type x Quadratic Time x zASQ | 1.53 | 2, 943265 | .216 | 0.001 |
| Image Type x Quadratic Time x AlcQF | 13.91 | 2, 943222 | **<.001** | 0.008 |
| *Note.*  Age is a grand-mean centered covariate representing each person's age in years.  Sex is a between-subject factor (2 levels: Female, Male).  Handedness is a between-subject factor (2 levels: Right-Hand Dominant, Not Right-Hand Dominant).  Linear Time is a person-centered covariate representing trial (i.e., observation) number relative to the total number of experimental trials in the task, rescaled such that -1 is the start of the task, 0 is the midpoint, and +1 is the end of the task.  Quadratic Time is the square of Linear Time.  AlcQF is a grand-mean centered covariate representing each person’s past year alcohol use quantity-frequency (Q-F) product score.  zASQ is a grand-mean centered covariate representing each person’s sex-stratified Alcohol Sensitivity Questionnaire (ASQ) score computed using the standardized person mean imputation method (Lee et al., 2015).  Degrees of freedom (*df*) were estimated using Satterthwaite’s method (Satterthwaite, 1941). | | | | |

| **Table S5**  *Model-Estimated Simple Slopes of Linear and Quadratic Time on P3 Mean Amplitude* | | | | |
| --- | --- | --- | --- | --- |
| Simple Slopes | *b* | *SE* | *z* | *p* |
| ***Base Model*** | | | | |
| Linear Time | | | | |
| AlcBev Cue^a^ | 1.20 | 0.07 | 16.30 | **<.001** |
| NADrink Cue^b^ | 1.47 | 0.07 | 19.75 | **<.001** |
| Neutral Cue^c^ | -0.06 | 0.06 | -1.01 | **.314** |
| Quadratic Time | | | | |
| AlcBev Cue^a^ | -1.17 | 0.12 | -9.71 | **<.001** |
| NADrink Cue^a^ | -1.29 | 0.12 | -10.61 | **<.001** |
| Neutral Cue^b^ | 0.37 | 0.09 | 4.23 | **<.001** |
| ***Hypothesis-Testing Model*** | | | | |
| Linear Time |  |  |  |  |
| LS |  |  |  |  |
| Alcohol Cue^a, d^ | 1.21 | 0.13 | 9.19 | **<.001** |
| NADrink Cue^b, e^ | 1.72 | 0.13 | 12.89 | **<.001** |
| Neutral Cue^b, f^ | -0.18 | 0.11 | 1.66 | 0.096 |
| HS |  |  |  |  |
| Alcohol Cue^a, d^ | 1.20 | 0.13 | 9.14 | **<.001** |
| NADrink Cue^a, g^ | 1.23 | 0.13 | 9.26 | **<.001** |
| Neutral Cue^b, f^ | 0.06 | 0.11 | 0.55 | 0.584 |
| LA |  |  |  |  |
| Alcohol Cue^a, d^ | 1.27 | 0.09 | 13.39 | **<.001** |
| NADrink Cue^b, e^ | 1.64 | 0.10 | 17.14 | **<.001** |
| Neutral Cue^c, f^ | -0.01 | 0.08 | 0.12 | 0.901 |
| HA |  |  |  |  |
| Alcohol Cue^a, d^ | 1.17 | 0.08 | 15.03 | **<.001** |
| NADrink Cue^b, e^ | 1.39 | 0.08 | 17.70 | **<.001** |
| Neutral Cue^c, f^ | -0.08 | 0.06 | 1.31 | 0.189 |
| Quadratic Time |  |  |  |  |
| LS |  |  |  |  |
| Alcohol Cue^a, d^ | -1.33 | 0.22 | 6.17 | **<.001** |
| NADrink Cue^a, e^ | -1.12 | 0.22 | 5.11 | **<.001** |
| Neutral Cue^b, f^ | 0.36 | 0.16 | 2.29 | **.022** |
| HS |  |  |  |  |
| Alcohol Cue^a, d^ | -1.02 | 0.21 | 4.76 | **<.001** |
| NADrink Cue^b, e^ | -1.46 | 0.22 | 6.69 | **<.001** |
| Neutral Cue^c, f^ | 0.38 | 0.16 | 2.43 | **.015** |
| LA |  |  |  |  |
| Alcohol Cue^a, d^ | -1.55 | 0.16 | 9.94 | **<.001** |
| NADrink Cue^a, e^ | -1.31 | 0.16 | 8.32 | **<.001** |
| Neutral Cue^b, f^ | 0.40 | 0.11 | 3.48 | **<.001** |
| HA |  |  |  |  |
| Alcohol Cue^a, g^ | -1.01 | 0.13 | 7.87 | **<.001** |
| NADrink Cue^b, e^ | -1.28 | 0.13 | 9.91 | **<.001** |
| Neutral Cue^c, f^ | 0.36 | 0.09 | 3.86 | **<.001** |
| *Note.*  Simple slopes of linear and quadratic time by cue type estimated from the indicated covariate-adjusted LMMs are shown. Within each set of simple slopes, matching superscript letters a/b/c indicate that the slopes are statistically similar and mismatched letters a/b/c indicate uncorrected *p*<.05 for the pairwise comparison of the slopes. Matching or mismatched superscript letters d/e/f/g are used to indicate similarity or dissimilarity between phenotypes.  HS = high sensitivity to alcohol. LS = low sensitivity to alcohol.  LA = light alcohol use. HA = heavy alcohol use.  Linear Time is a person-centered covariate representing trial (i.e., observation) number relative to the total number of experimental trials in the task, rescaled such that -1 is the start of the task, 0 is the midpoint, and +1 is the end of the task.  Quadratic Time is the square of Linear Time.  For the simple slopes by cue type listed under Hypothesis-Testing Models, LMM-estimated *b* and *SE* were derived: (i) once holding zASQ at *z* = -1 SD, which corresponds to HS phenotypes; (ii) once holding zASQ at *z* = +1 SD, which corresponds to LS phenotypes; (iii) once holding AlcQF at its lower quartile, which corresponds to LA phenotypes; and (iv) once holding AlcQF at its upper quartile, which corresponds to HA phenotypes. Note that, for completeness, quadratic trends over time by cue type are presented for LS and HS phenotypes despite non-significant *F*-test for the zASQ x Quadratic Time x Image Type interaction effect. | | | | |

**Figure S1.** Scalp topography of the event-related potential (ERP) response to alcohol/nonalcohol picture oddball stimuli and neutral picture standard stimuli

**
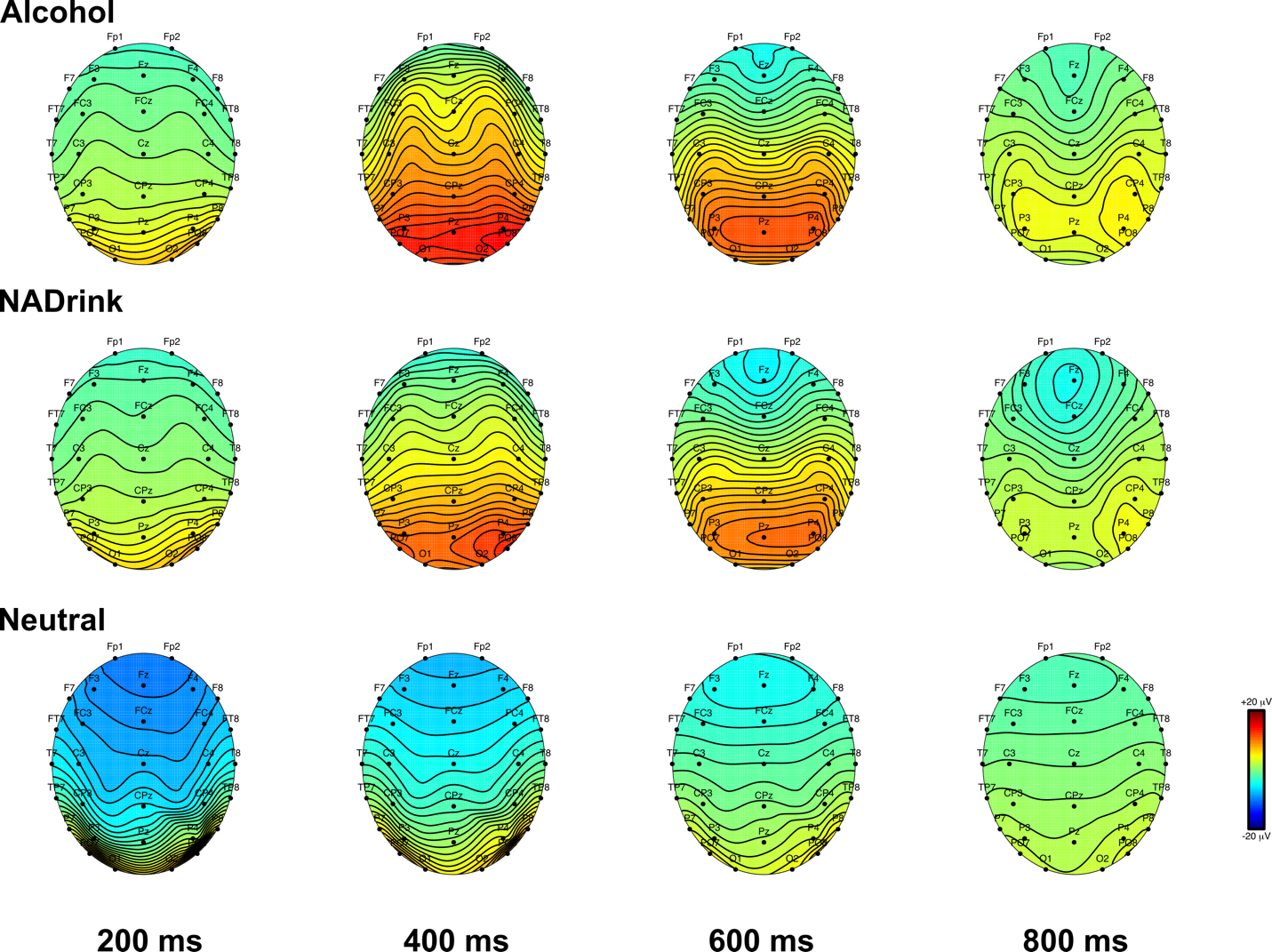
**

*Note.* Picture onset occurs at 0 ms. Picture offset occurs at 1000 ms. Alcohol = alcohol beverage pictures. NADrink = nonalcohol drink pictures. Neutral = affectively neutral pictures. Positivity over occipitoparietal scalp for Alcohol and NADrink images types visible in the scalp maps at 400 and 600 ms post-stimulus corresponds to the P3 component. The P3 was maximal over a cluster of 9 occipito-parietal electrodes (PZ, P3, P4, P7, P8, PO7, PO8, O1, O2). Data represent *N* = 287 participants.

**Figure S2.** Within-session trajectory of P3 mean amplitude (µV) for alcohol/nonalcohol picture oddball stimuli and neutral picture standard stimuli.


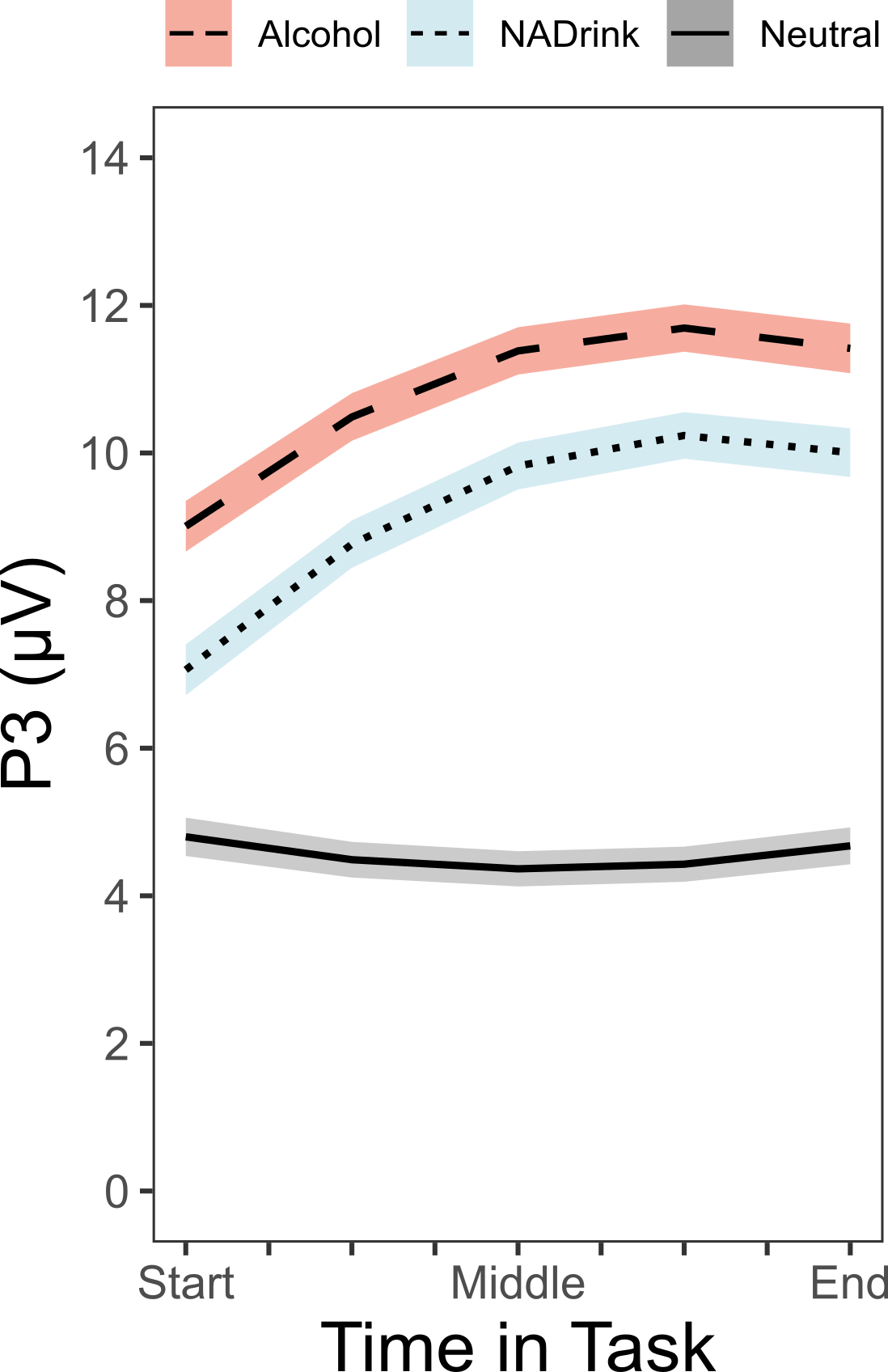


*Note*. Alcohol = alcohol beverage pictures. NADrink = nonalcohol drink pictures. Neutral = affectively neutral pictures. Start = first artifact-free trial for each person. Middle = artifact-free trial that bisects each person's set of artifact-free trials. End = final artifact-free trial for each person. Thin line at the center of each colored ribbon represents the covariate-adjusted LMM-estimated *M* P3 score at different relative times in the picture viewing task and the thickness of each colored ribbon represents the covariate-adjusted LMM-estimated ±1 *SE*.

**Figure S3.** Differences in mean amplitude (µV) of P3 responses to alcohol and nonalcohol picture stimuli at different times in the task.


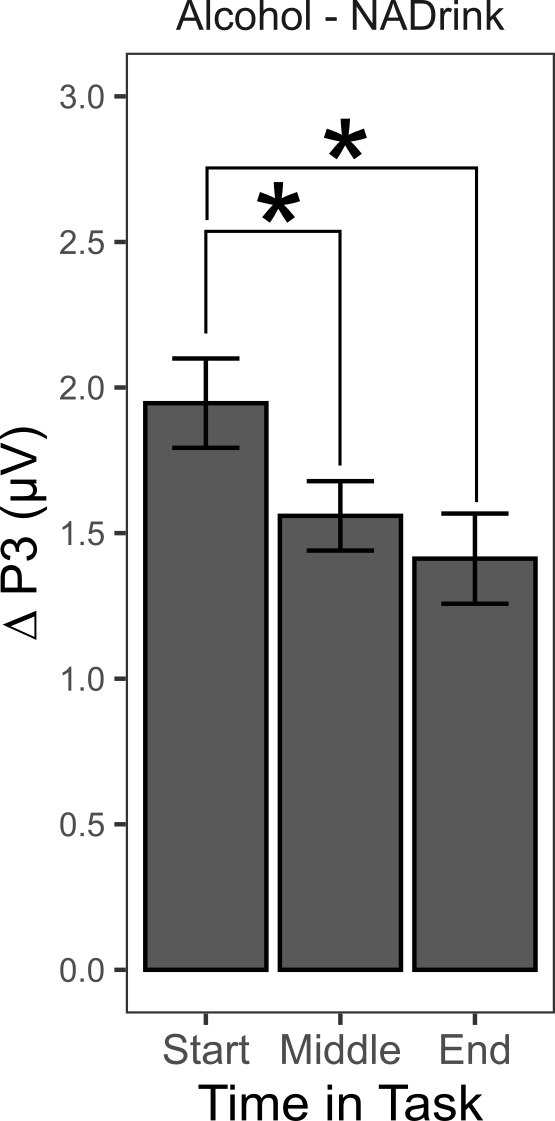


*Note*. Alcohol = alcohol beverage pictures. NADrink = nonalcohol drink pictures. Start = first artifact-free trial for each person. Middle = artifact-free trial that bisects each person's set of artifact-free trials. End = final artifact-free trial for each person. Covariate-adjusted LMM-estimated *M* P3 difference scores shown at different relative times in the picture viewing task. Error bars = ±1 *SE*. * = *p* < .05.

**Figure S4.** Differences in mean amplitude (µV) of P3 responses to alcohol or nonalcohol beverage relative to neutral picture stimuli at different times in the task.

**
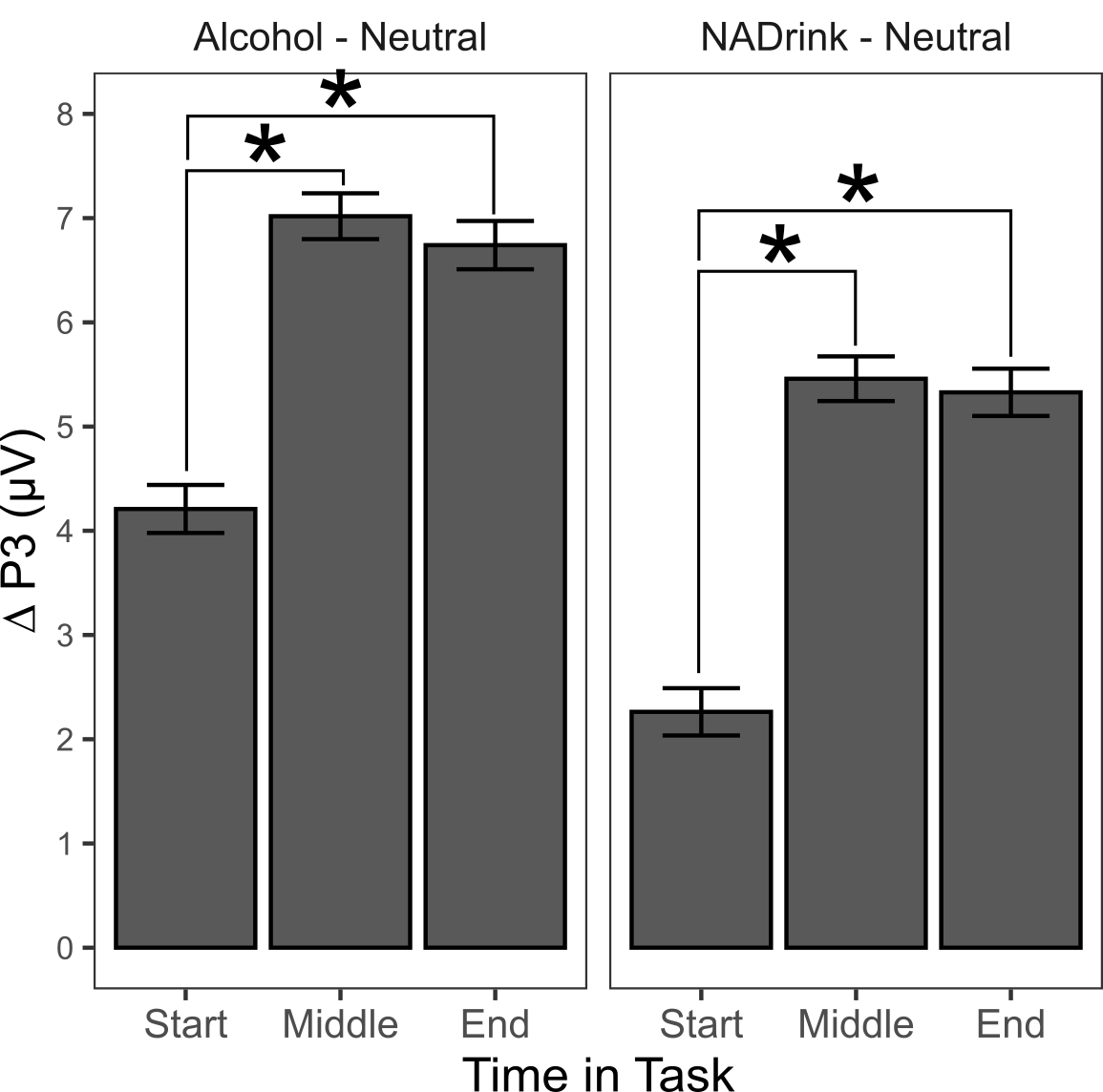
**

*Note.* Alcohol = alcohol beverage pictures. NADrink = nonalcohol drink pictures. Neutral = affectively neutral pictures. Start = first artifact-free trial for each person. Middle = artifact-free trial that bisects each person's set of artifact-free trials. End = final artifact-free trial for each person. Covariate-adjusted LMM-estimated *M* P3 difference scores shown at different relative times in the picture viewing task. Error bars = ±1 *SE*. * = *p* < .05.

**Figure S5.** Differences in mean amplitude (µV) of P3 responses to alcohol or nonalcohol beverage relative to neutral picture stimuli at different times in the task as a function of alcohol sensitivity.

**
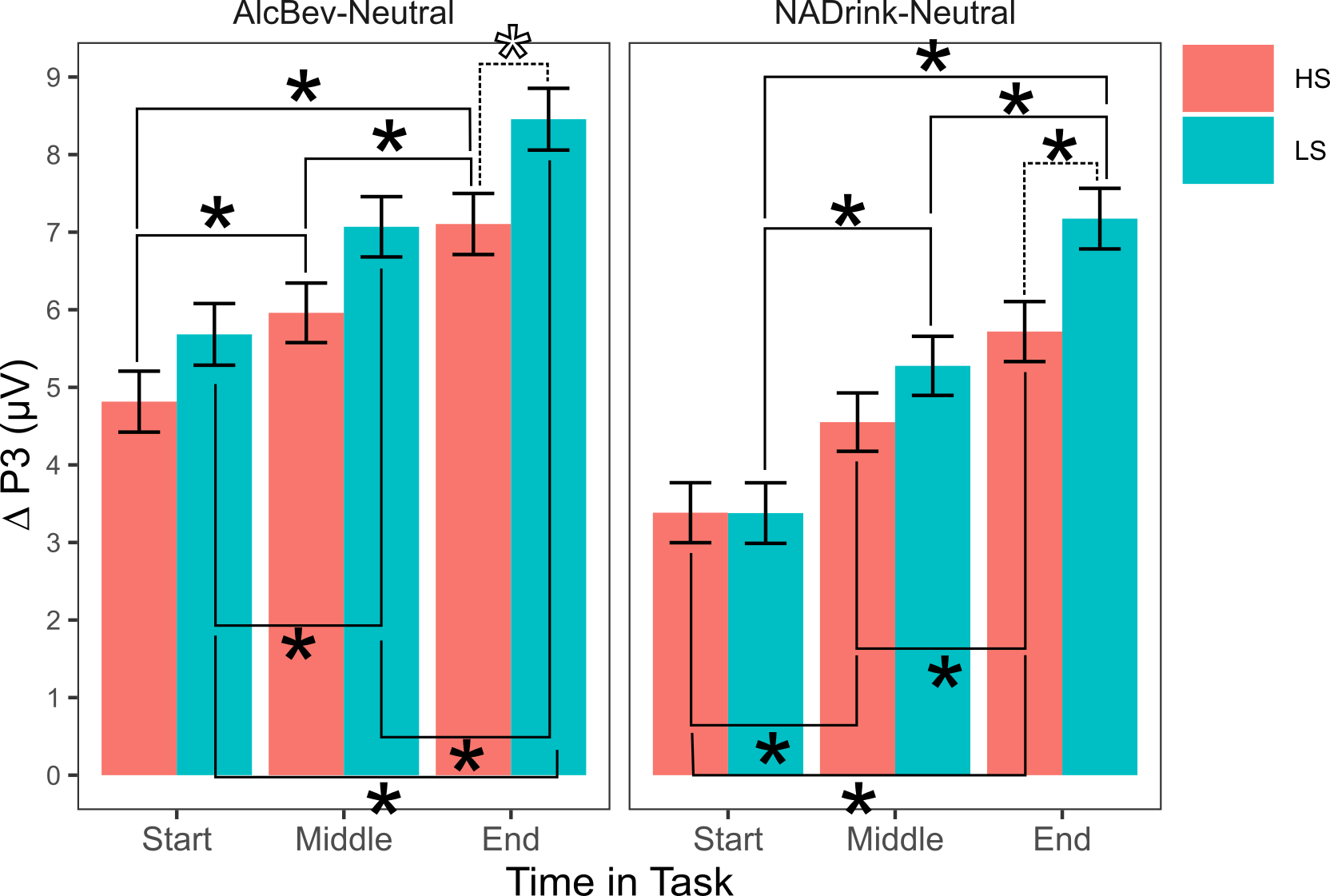
**

*Note*. Alcohol = alcohol beverage pictures. NADrink = nonalcohol drink pictures. Neutral = affectively neutral pictures. Start = first artifact-free trial for each person. Middle = artifact-free trial that bisects each person's set of artifact-free trials. End = final artifact-free trial for each person. Covariate-adjusted LMM-estimated *M* P3 difference scores shown at different relative times in the picture viewing task. Error bars = ±1 *SE*. LMM-estimated *M* ± *SE* were derived twice: once holding zASQ at *z* = -1 SD, which corresponds to higher sensitivity (HS) phenotypes, and once holding zASQ at *z* = +1 SD, which corresponds to lower sensitivity (LS) phenotypes. * = *p* < .05. Dashed line = within-timepoint, phenotype comparison. Solid line = within-phenotype, timepoint comparisons.

**Figure S6.** Within-session trajectory of P3 mean amplitude (µV) for alcohol/nonalcohol picture oddball stimuli and neutral picture standard stimuli as a function of past year alcohol use.


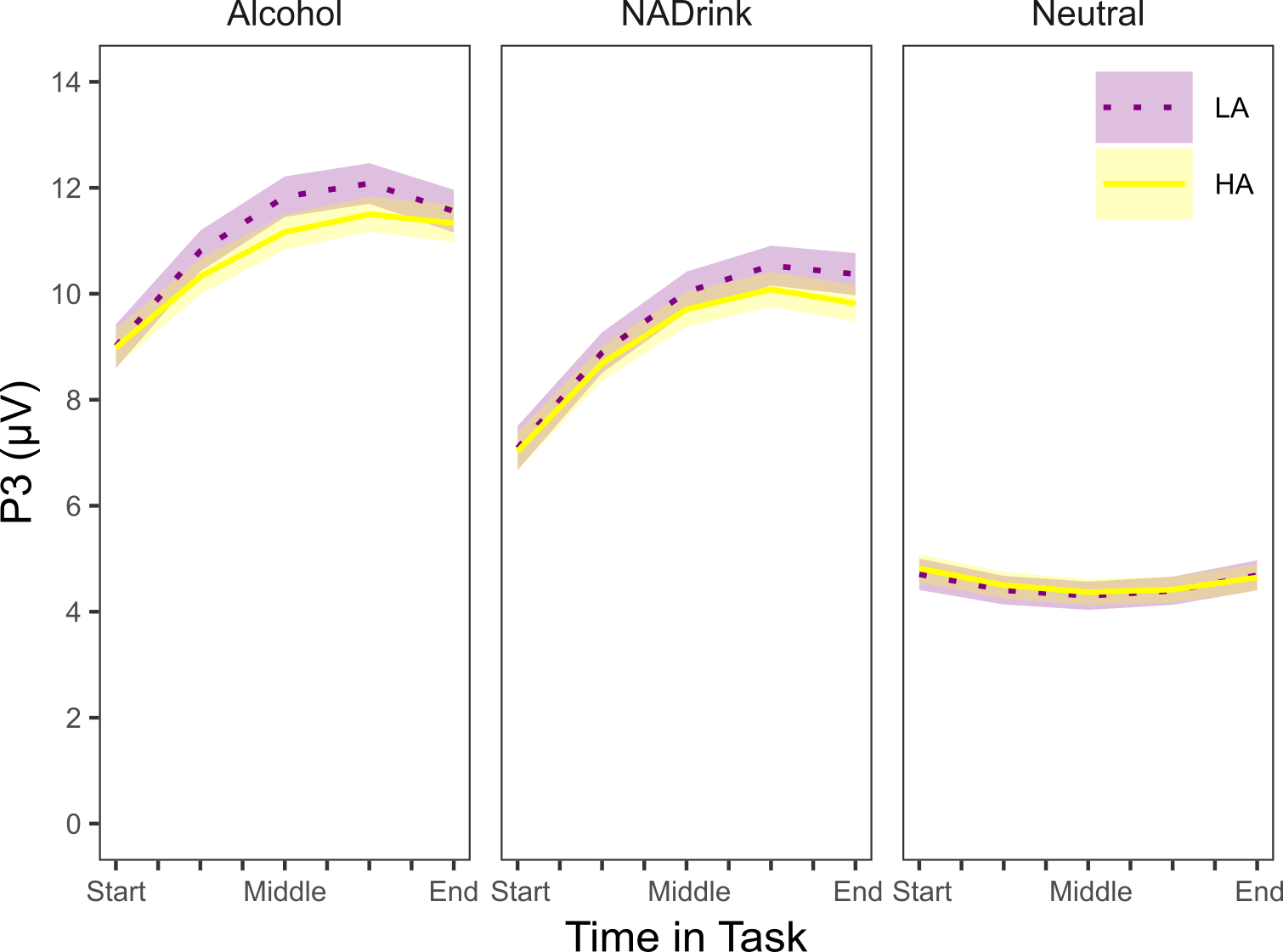


*Note*. Alcohol = alcohol beverage pictures. NADrink = nonalcohol drink pictures. Neutral = affectively neutral pictures. Start = first artifact-free trial for each person. Middle = artifact-free trial that bisects each person's set of artifact-free trials. End = final artifact-free trial for each person. Thin line at the center of each colored ribbon represents the covariate-adjusted LMM-estimated *M* P3 score at different relative times in the picture viewing task and the thickness of each colored ribbon represents the covariate-adjusted LMM-estimated ±1 *SE*. LMM-estimated *M* ± *SE* were derived twice: once holding AlcQF at its lower quartile, which corresponds to lighter alcohol use (LA) phenotypes, and once holding AlcQF at its upper quartile, which corresponds to heavier alcohol use (HA) phenotypes. AlcQF was the grand-mean centered past year typical alcohol use quantity (drinks per drinking day) x frequency (drinking days per week) composite score.

**Figure S7.** Differences in P3 mean amplitude (µV) among alcohol, nonalcohol, and neutral picture stimuli at different times in the task as a function of past year alcohol use.


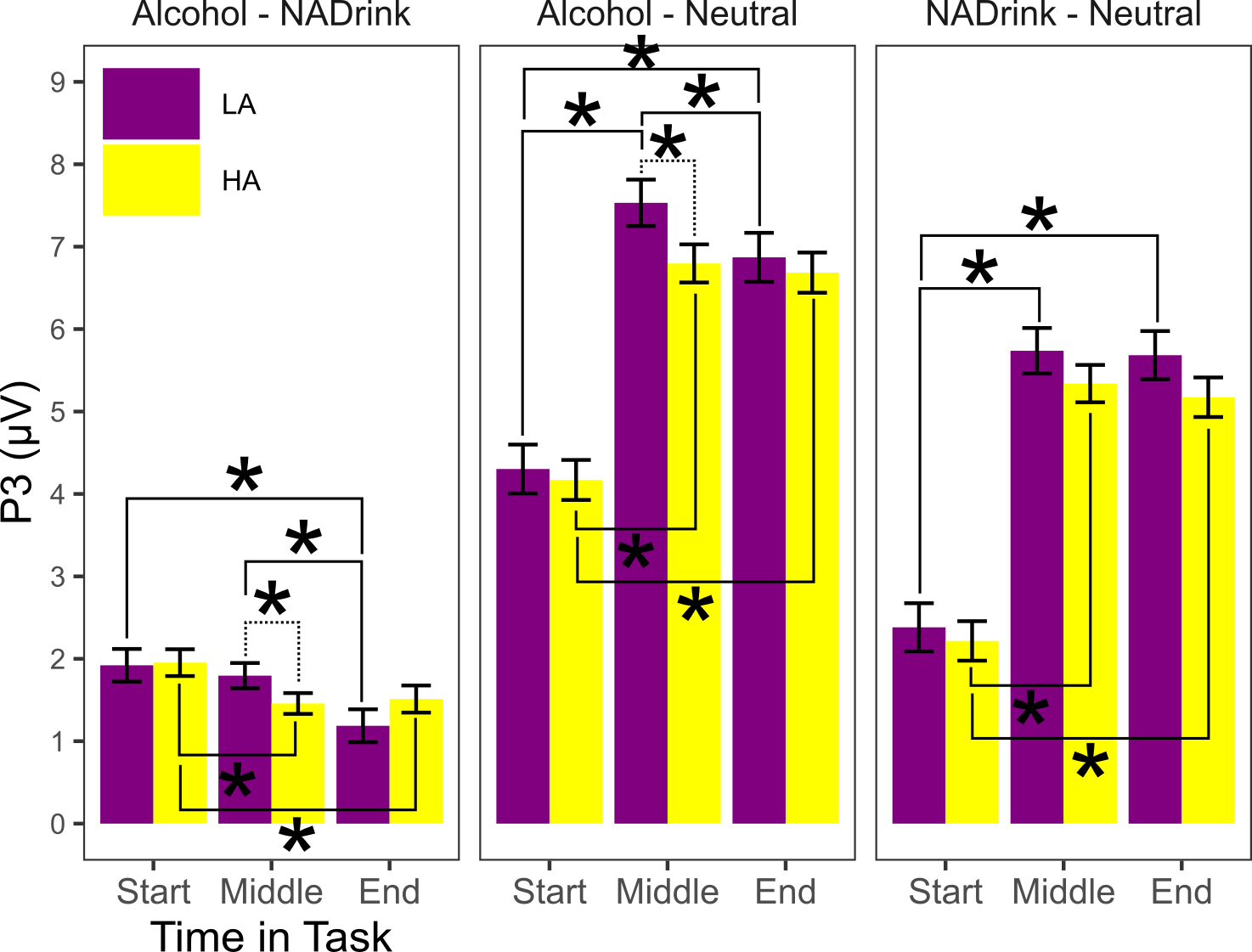


*Note*. Alcohol = alcohol beverage pictures. NADrink = nonalcohol drink pictures. Neutral = affectively neutral pictures. Start = first artifact-free trial for each person. Middle = artifact-free trial that bisects each person's set of artifact-free trials. End = final artifact-free trial for each person. Covariate-adjusted LMM-estimated *M* P3 difference scores shown at different relative times in the picture viewing task. Error bars = ±1 *SE*. LMM-estimated *M* ± *SE* were derived twice: once holding AlcQF at its lower quartile, which corresponds to lighter alcohol use (LA) phenotypes, and once holding AlcQF at its upper quartile, which corresponds to heavier alcohol use (HA) phenotypes. AlcQF was the grand-mean centered past year typical alcohol use quantity (drinks per drinking day) x frequency (drinking days per week) composite score. * = *p* < .05. Dashed line = within-timepoint, phenotype comparison. Solid line = within-phenotype, timepoint comparisons.

1. For participants, screening survey compensation was issued at the same time as compensation for session 1. Everyone else who completes the screening survey but does not go on to participate in the study is entered into a lottery to receive one of 30 electronic giftcards ($15 each) when the study ends. [↑](#footnote-ref-1)
2. IAPS image codes: 1122, 1350, 1616, 1670, 1675, 1903, 1908, 1935, 1947, 5040, 5120, 5130, 5390, 5395, 5471, 5500, 5510, 5520, 5530, 5531, 5532, 5533, 5534, 5535, 5740, 6150, 7002, 7003, 7004, 7006, 7010, 7011, 7012, 7014, 7016, 7017, 7018, 7019, 7020, 7021, 7025, 7026, 7030, 7032, 7033, 7034, 7036, 7037, 7038, 7039, 7040, 7041, 7043, 7045, 7050, 7052, 7053, 7055, 7056, 7059, 7090, 7140, 7161, 7175, 7180, 7205, 7217, 7224, 7234, 7287, 7290, 7491, 7495, 7705, 7950, 9360, 9469. [↑](#footnote-ref-2)
3. ABPS image codes: SDC10695, SDC10709, SDC10716, SDC10917, SDC11010, SDC11069, SDC10744, SDC10804, SDC10808, SDC10815, SDC10821, SDC10825, SDC10836, SDC10858, SDC10946, SDC10967. [↑](#footnote-ref-3)
4. Budweiser can, Coors Light can, Natural Light can, and Jack Daniel’s bottle alongside a filled shot glass. [↑](#footnote-ref-4)
5. Although it is extremely unlikely participants perceived a difference, it is important to note that due to an error during task implementation in E-Prime, for about half the participants, the first experimental trial block utilized a pseudo-random stimulus sequence (as in our previous work (Bartholow et al., 2007, 2010, 2018; Martins et al., 2019) with an oddball stimulus occurring after every 3, 4, 5, or 7 standard stimuli) that was yoked across participants whereas the second experimental trial block utilized the intended random selection process. We probed the data for any systematic differences owing to block 1 stimulus sequence type (random vs. yoked), but there were neither significant between-subject main effects of block 1 stimulus sequence type nor significant interactions with the within-subject effect of picture type, on P3 mean amplitudes, *F* < 1, *p* ≥ .20. [↑](#footnote-ref-5)
